# Carbohydrate metabolic systems present on genomic islands are lost and gained in *Vibrio parahaemolyticus*

**DOI:** 10.1186/s12866-019-1487-6

**Published:** 2019-05-27

**Authors:** Abish Regmi, Ethna Fidelma Boyd

**Affiliations:** Department of Biological Sciences, University of Delaware, 341 Wolf Hall, Newark, DE 19716 USA

**Keywords:** Sodium/galactose transporter SGLT, l-rhamnose, l-arabinose, Entner-Doudoroff aldolase (EDA), Metabolism islands, Citrate fermentation, Tn7-like transposon, CRISPR-Cas systems

## Abstract

**Background:**

Utilizing unique carbohydrates or utilizing them more efficiently help bacteria expand and colonize new niches. Horizontal gene transfer (HGT) of catabolic systems is a powerful mechanism by which bacteria can acquire new phenotypic traits that can increase survival and fitness in different niches. In this work, we examined carbon catabolism diversity among *Vibrio parahaemolyticus*, a marine species that is also an important human and fish pathogen.

**Results:**

Phenotypic differences in carbon utilization between *Vibrio parahaemolyticus* strains lead us to examine genotypic differences in this species and the family *Vibrionaceae* in general. Bioinformatics analysis showed that the ability to utilize d-galactose was present in all *V. parahaemolyticus* but at least two distinct transporters were present; a major facilitator superfamily (MFS) transporter and a sodium/galactose transporter (SGLT). Growth and genetic analyses demonstrated that SGLT was a more efficient transporter of d-galactose and was the predominant type among strains. Phylogenetic analysis showed that d-galactose gene *galM* was acquired multiples times within the family *Vibrionaceae* and was transferred between distantly related species. The ability to utilize d-gluconate was universal within the species. Deletion of *eda* (VP0065), which encodes aldolase, a key enzyme in the Entner-Doudoroff (ED) pathway, reached a similar biomass to wild type when grown on d-gluconate as a sole carbon source. Two additional *eda* genes were identified, VPA1708 (*eda2*) associated with a d-glucuronate cluster and VPA0083 (*eda3*) that clustered with an oligogalacturonide (OGA) metabolism cluster. EDA2 and EDA3 were variably distributed among the species. A metabolic island was identified that contained citrate fermentation, l-rhamnose and OGA metabolism clusters as well as a CRISPR-Cas system. Phylogenetic analysis showed that CitF and RhaA had a limited distribution among *V. parahaemolyticus*, and RhaA was acquired at least three times. Within *V. parahaemolyticus*, two different regions contained the gene for L-arabinose catabolism and most strains had the ability to catabolism this sugar.

**Conclusion:**

Our data suggest that horizontal transfer of metabolic systems among *Vibrionaceae* is an important source of metabolic diversity. This work identified four EDA homologues suggesting that the ED pathway plays a significant role in metabolism. We describe previously uncharacterized metabolism islands that were hotspots for the gain and loss of functional modules likely mediated by transposons.

**Electronic supplementary material:**

The online version of this article (10.1186/s12866-019-1487-6) contains supplementary material, which is available to authorized users.

## Background

*Vibrio parahaemolyticus* is a Gram-negative, moderate halophile that is ubiquitous in the marine and estuarine environments, present in sediments, shellfish and zooplankton [1–4]. Consumption of raw and undercooked shellfish is the primary route of *V. parahaemolyticus* into the human host, where it is the leading cause of bacterial seafood-related gastroenteritis worldwide [5, 6]. According to CDC, in the United States alone 45,000 cases of *V. parahaemolyticus* illnesses are estimated each year [7].

In 1996, a highly virulent *V. parahaemolyticus* serogroup O3:K6 strain emerged in Asia and has since disseminated globally [5]. This pandemic clone was sequenced, strain RIMD2210633, and two type three secretion systems (T3SS), one on each chromosome, designated T3SS-1 and T3SS-2 were identified [8]. T3SS-1 is present in all isolates and is ancestral to the species, whereas T3SS-2 is present on a pathogenicity island and is found in pathogenic strains, with a number of variants of this region described [8–12]. Studies have identified at least three variant T3SS-2 systems; T3SS-2α present on chromosome 2 and the non-homologous T3SS-2β system present on chromosome 1 or chromosome 2, and T3SS-2γ on chromosome 2 that is closely related to T3SS2β [12–14]. T3SS-2γ was identified in strains that are members of the clonal complex ST631, a recent pathogenic clone to emerge in the Northeast USA [12, 15, 16]. A recent study has proposed that T3SS-2α is part of a novel Tn-7 like transposon that has co-opted a mini CRISPR-Cas system to potentially mobilize the entire region [17].

*Vibrio parahaemolyticus* is a marine species that has adapted and evolved to colonize and establish niches in the intestine of humans as well as fish and shellfish species. In these environments, the bacterium must compete with endogenous microbiota and other pathogenic bacteria for available nutrients. Freter’s nutrient-niche hypothesis, proposes that any species of bacteria should utilize at least one limiting nutrient better than another species for successful colonization [18]. Previous work has shown that like most *Vibrio* species, *V. parahaemolyticus* can catabolize N-acetyl-d-glucosamine, a monomer of chitin highly prevalent in the marine environment. Using a phenotypic array of 190 carbon sources, it was shown that *V. parahaemolyticus* could utilize 71 different carbon sources under static growth conditions [19]. Studies have shown that strains have also adapted to utilize mouse intestinal mucus and mucus sugars l-arabinose, d-galactose, d-gluconate, d-glucuronate, d-mannose, d-glucosamine, and d-ribose as sole carbon sources [19, 20]. Previously, it was demonstrated that disruption of metabolism global regulation can have a profound effect on fitness [19, 20]. For example, deletion of the global transcription factor sigma 54 encoded by *rpoN* enhanced in vivo fitness and this correlated with increased rates of carbon metabolism [20]. Whereas deletion of the quorum sensing response regulator *luxO* inhibited carbon metabolism and caused growth defects [19].

In the present study, differences among *V. parahaemolyticus* strains in their ability to utilize different carbohydrate sources was determined using bioinformatics, phenotypic and genetic analyses. First, phenotypic differences between a clinical strain RIMD2201633 and an environmental strain UCM-V493 were examined and significant differences in growth on many carbon sources were found, of note was d-galactose utilization. Both strains had identical catabolic gene clusters but each strain had a different galactose transporter*;* a major facilitator superfamily (MFS) transporter in RIMD2210633 and a sodium/galactose transporter (SGLT) in UCM-V493. Growth analysis revealed that RIMD2201633 had a significant lag phase when grown in d-galactose compared to UCM-V493. Genetic complementation suggested that SGLT is a high efficiency d-galactose transporter, which was the predominant transporter present among *V. parahaemolyticus* strains. Our analysis showed that d-galactose utilization is phylogenetically widespread among the *Vibrionaceae*, present in divergent genera and species but not highly prevalent. A d-gluconate cluster was near universal among *V. parahaemolyticus,* but had a restricted distribution within the *Vibrionaceae*. Interestingly, deletion of *eda1* (VP0065), that encodes 2-keto-3-deoxy-6-phosphate-gluconate (KDPG) aldolase (EDA), a key enzyme in the Entner–Doudoroff (ED) pathway, did not inhibit growth on d-gluconate. Bioinformatics identified two additional putative KDPG aldolases (*eda3* (VPA0083) and *eda2* (VPA1708)) in RIMD2210633. All three genes were induced in the presence of d-gluconate. EDA2 and EDA3 were variably distributed among *V. parahaemolyticus* and EDA1 was present in all strains Genome comparative analysis identified a 73-kb to 166-kb metabolic island that contained a citrate fermentation cluster, an l-rhamnose cluster and an oligogalacturonide metabolism cluster with an EDA homologue. This island also contained a type I-F CRISPR-Cas system and a Type IVb pilus. These different functional modules were flanked by transposase genes within the island suggesting a mechanism of acquisition. Two different regions contained l-arabinose metabolism clusters and most *V. parahaemolyticus* strains had the ability to catabolism this sugar.

## Results

### Different d-galactose transporters associated with different *V. parahaemolyticus* isolates

**Fig. 1:**
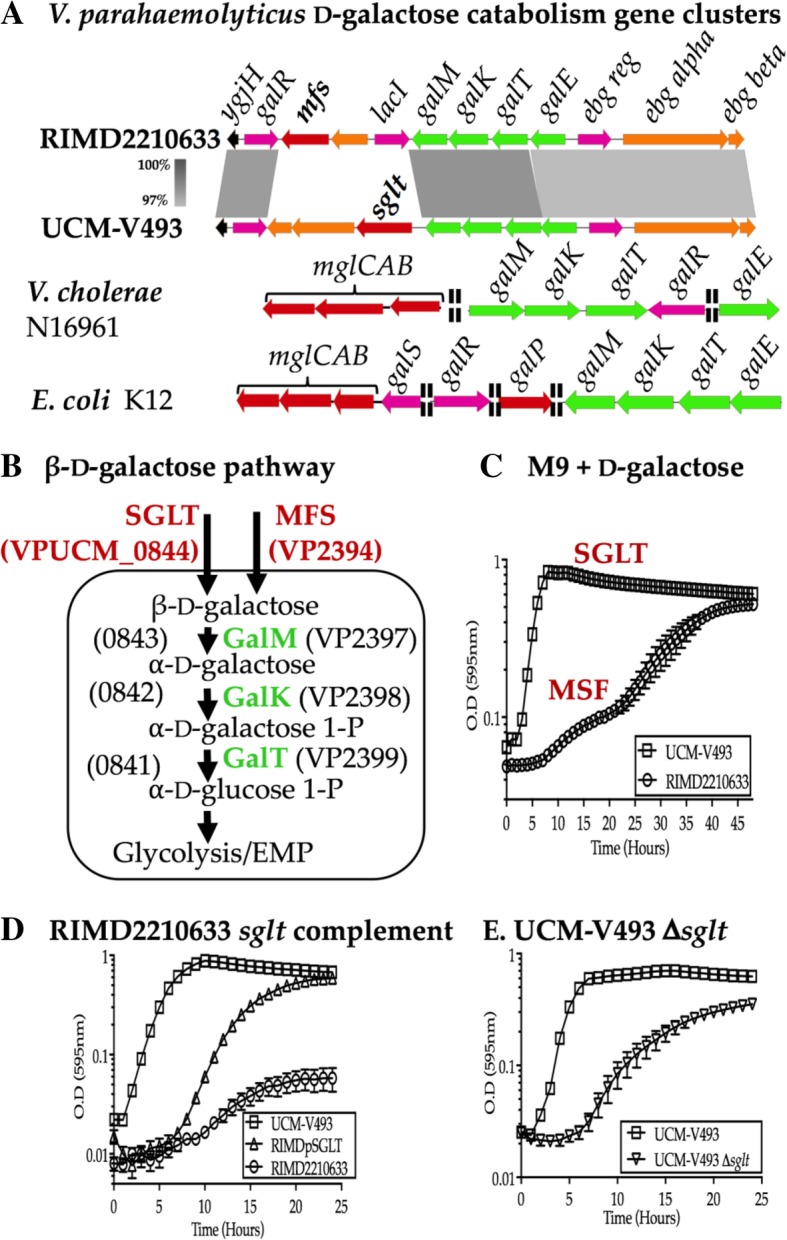
d-galactose utilization gene cluster. **a**. Comparative genomic analysis of the d-galactose catabolism and transport region from RIMD2210633, UCM-V493, *V. cholerae* N16961 and *E. coli* MG1655. Gray shade, region of nucleotide homology. **b**. d-galactose utilization pathway showing enzymes and open reading frames (ORFs) designations in RIMD2210633 and UCM-V493. **c**. RIMD2210633 (open circles) and UCM-V493 (open squares) were grown aerobically at 37 °C for 48 h in M9 + 10 mM d-galactose. **d**. Growth analysis of RIMD2210633, UCM-V493 and RIMDpSGLT (the *sglt* gene (ORF VPUCM_0844) was cloned into RIMD2210633) in M9 + 10 mM d-galactose after 24 h. **e**. Growth analysis of UCM-V493 and Δ*sglt* at 37 °C in M9 + 10 mM d-galactose

Comparative phenotypic growth analysis of RIMD2210633, a clinical strain and UCM-V493, an environmental strain in different carbon sources uncovered significant differences between the two strains in their ability to use different carbon sources (Additional file 1: Table S1). In additionto overall biomass differences between the strains, there were also differences in the ability to use certain carbon sources. UCM-V493 was unable to grow in L-arabinose and D-glucuronate, whereas RIMD2210633 was unable to grow in Tween 20, glycyl-L-glutamic acid, propionic acid and chondroitin sulfate C as sole carbons sources (Additional file 1: Table S1). One interesting difference noted between the strains was their ability to utilize d-galactose since both strains contained the catabolic genes in a highly homologous operon *galETKM*. d-galactose, a hexose, is a component of intestinal mucus and was demonstrated to be an essential carbohydrate for in vivo fitness of *E. coli* [21]. In *E. coli,* multiple galactose transporters were identified with at least two critical for galactose transport, MglBAC, an ATP binding cassette (ABC) type transporter, and GalP, a H+/symporter type transporter [22]. In *V. parahaemolyticus,* all strains contain the galactose catabolic cluster, however, no strain contained a homologue of the MglBAC or the GalP type transporters (Fig. 1a and b). A major facilitator superfamily (MFS) transporter was identified in RIMD2210633 and a novel sodium/galactose transporter (SGLT) transporter was present in UCM-V493 adjacent to the *gal* genes (Fig. 1a and Additional file 1: Table S2). The SGLT transporter shares 32% sequence identity (60% similarity) with that of human SGLT1 (hSGLT1) [23].

Further examination of the growth patterns of RIMD2210633 and UCM-V493 in M9 supplemented with d-galactose as a sole carbon source showed RIMD2210633 had a much longer lag phase and significantly lower biomass compared to UCM-V493 after 24 h (Fig. 1c). After 48 h, RIMD2210633 reached a similar biomass to UCM-V493 (Fig. 1c), which suggested that there were differences in galactose transport efficiency between the two strains. To examine this further, we complemented RIMD2210633 with the *sglt* gene present on an expression plasmid and examined growth in M9 d-galactose. RIMDpSGLT showed a significant reduction in lag phase indicating more efficient uptake of d-galactose (Fig. 1d). To further investigate the role of SGLT in UCM-V493, a *sglt* deletion mutant was constructed. Growth analysis of the Δ*sglt* mutant in M9 d-galactose showed the mutant had a longer lag phase (around 5-h lag) with a significantly lower biomass compared to wild type (Fig. 1e). This data demonstrated that SGLT is a major d-galactose transporter in UCM-V493 and that this strain harbors an additional uncharacterized d-galactose transporter. A similar finding was demonstrated in an *E. coli galP/ mgl* double mutant, which also showed growth in d-galactose. In that study, the authors showed that uptake in the double mutant was due to facilitated diffusion via PtsG, a glucose specific phosphotransferase system (PTS) permease [24].

Next, we determined the distribution of each of the d-galactose transporters within *V. parahaemolyticus*. To accomplish this, we first examined a subset of 48 strains representing pathogenic and environmental strains whose genome sequence was available in the NCBI genome database **(**Additional file 1: Table S2 and Table S3). The d-galactose MSF transporter was present in a range of pathogenic isolates recovered over 60 years from different continents. The MFS transporter was also the common type found in non-pathogenic strains, whereas the SGLT system was present mainly within pathogenic strains recovered in the 2000s **(**Additional file 1: Table S2). Examination of the entire *V. parahaemolyticus* genome database (> 650 genomes) showed that two thirds of strains contained SGLT and approximately a third of strains contained an MFS type.

Interestingly, the SGLT transporter was present in all strains that contained T3SS-2γ and further analysis showed that these strains lacked l-arabinose and d-glucuronate metabolism clusters (Additional file 1: Table S2). Strains that contain T3SS-2γ belong to the newly emerged pathogenic clone ST631 identified in the USA in the late 2000s [12, 15, 16]. One could speculate that acquisition of a highly efficient d-galactose transporter could give strains a competitive advantage in a low d-galactose niche or outcompete other species or strains in a d-galactose replete environment.

### Phylogenetic analysis of d-galactose catabolism and transporter proteins

**Fig. 2:**
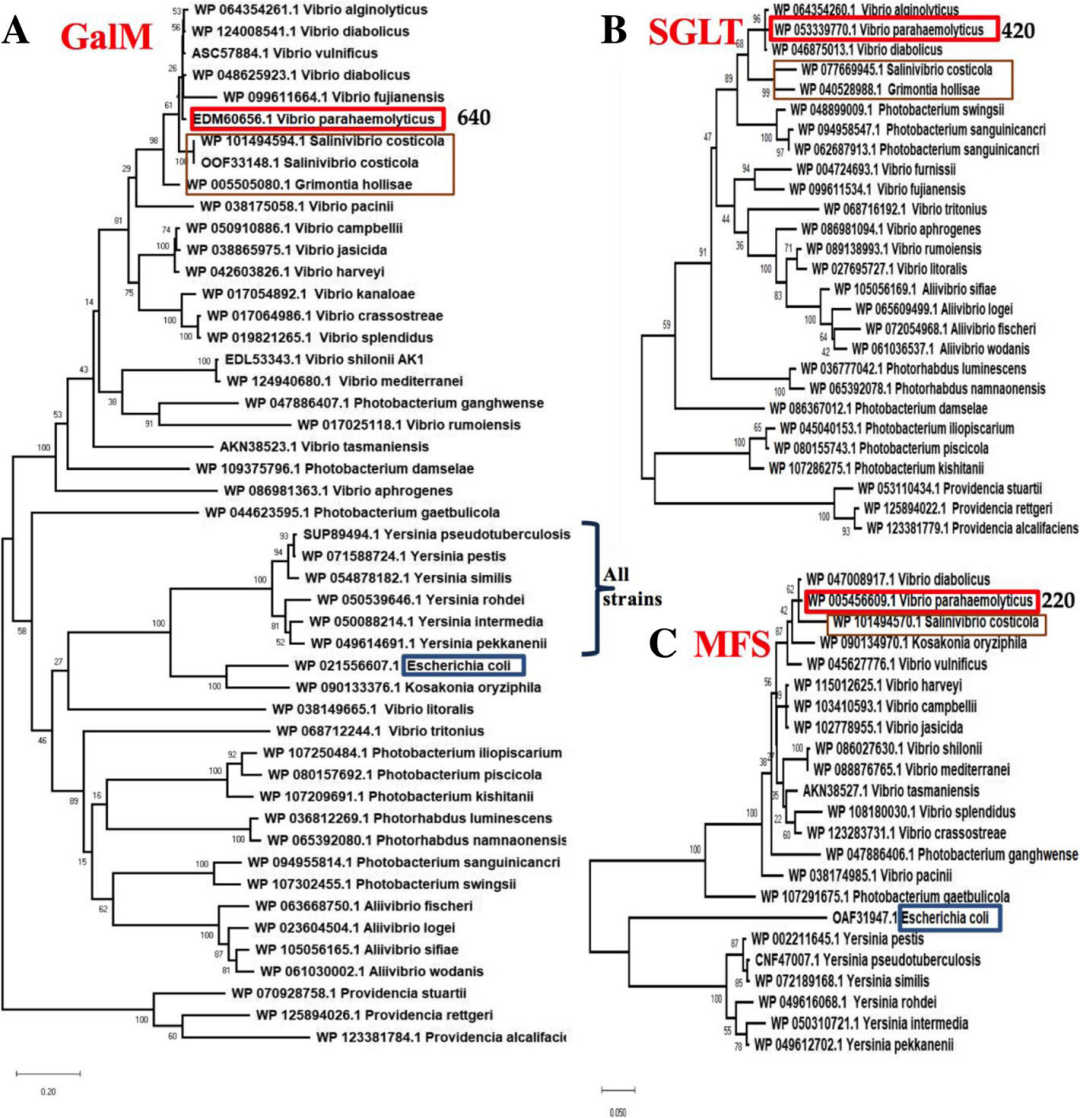
Phylogenetic analysis of **a**. GalM, **b**. MFS, and **c**. SGLT. Homologues of GalM, SGLT and MFS proteins from members of the *Vibrionaceae* were obtained from the NCBI genome database. The evolutionary history of each protein was inferred using the Neighbor-Joining method [26]. The percentage of replicate trees in which the associated taxa clustered together in the bootstrap test (1000 replicates) are shown next to the branches [27]. Each tree is drawn to scale, with branch lengths in the same units as those of the evolutionary distances used to infer the phylogenetic tree. The evolutionary distances were computed using the Dayhoff matrix based method [28] and are in the units of the number of amino acid substitutions per site. The rate variation among sites was modeled with a gamma distribution (shape parameter = 5). All ambiguous positions were removed for each sequence pair (pairwise deletion option). Evolutionary analyses were conducted in MEGA X [29]

In order to determine the evolutionary history of galactose catabolism and transport, we reconstructed the phylogeny of GalM, SGLT and MSF proteins among *Vibrio* species. Proteins representing homologues within the *Vibrionaceae* were aligned using ClustalW and phylogenetic trees were constructed by the neighbor-joining method [25, 26] (Fig. 2). Within the Campbellii clade, which consists of *V. campbellii, V. harveyi, V. alginolyticus, V. parahaemolyticus, V. diabolicus* and *V. jasicida*, all GalM proteins clustered together on the tree (Fig. 2a). However, nested within this cluster was GalM proteins from *Salinivibrio costicola* and *Grimontia hollisae*, which are distantly related to members of the Campbellii clade, which suggests recent horizontal transfer between these groups. In addition, the GalM protein from *Alliivibrio* and *Photobacterium* species clustered divergently from most *Vibrio* species and were more closely related to the GalM from *Yersinia* and *E. coli*. These data suggest very different evolutionary origins for d-galactose clusters among the *Vibrionaceae* and d-galactose utilization is phylogenetically widespread but not highly prevalent (Fig. 2a). Phylogenetic analysis of the SGLT protein associated with the *gal* operon in *V. parahaemolyticus* clustered closely with SGLT from *V. alginolyticus* and *V. diabolicus* strains and highly related to these was SGLT from *Salinivibrio costicola* and *Grimontia hollisae* (Fig. 2b)*.* SGLT was also the predominant transporter present in strains of *Alliivibrio* and *Photobacterium*, although the SGLT protein from *Photobacterium* species was more closely related to SGLT from species of *Providencia* (Fig. 2b). The MFS protein from *V. parahaemolyticus* clustered together with MFS protein from *V. diabolicus* and *S. costicola* indicating that in these species, similar to *V. parahaemolyticus,* different transporters can be associated with the d-galactose catabolism cluster depending on the strain (Fig. 2c). Other members of the Campbellii group (*V. campbellii, V. harveyi, V. jasicida*) contained an MFS protein and clustered tightly together (short branch lengths). Distantly related to these was MFS proteins from *Yersinia* and *E. coli* present on separate divergent branches. Similar to the GalM protein, the SGLT and MFS proteins clustering patterns suggest multiple acquisition events.

### Three Entner-Doudoroff (ED) aldolases (EDA) (VP0065, VPA0083 and VPA1708)

**Fig. 3:**
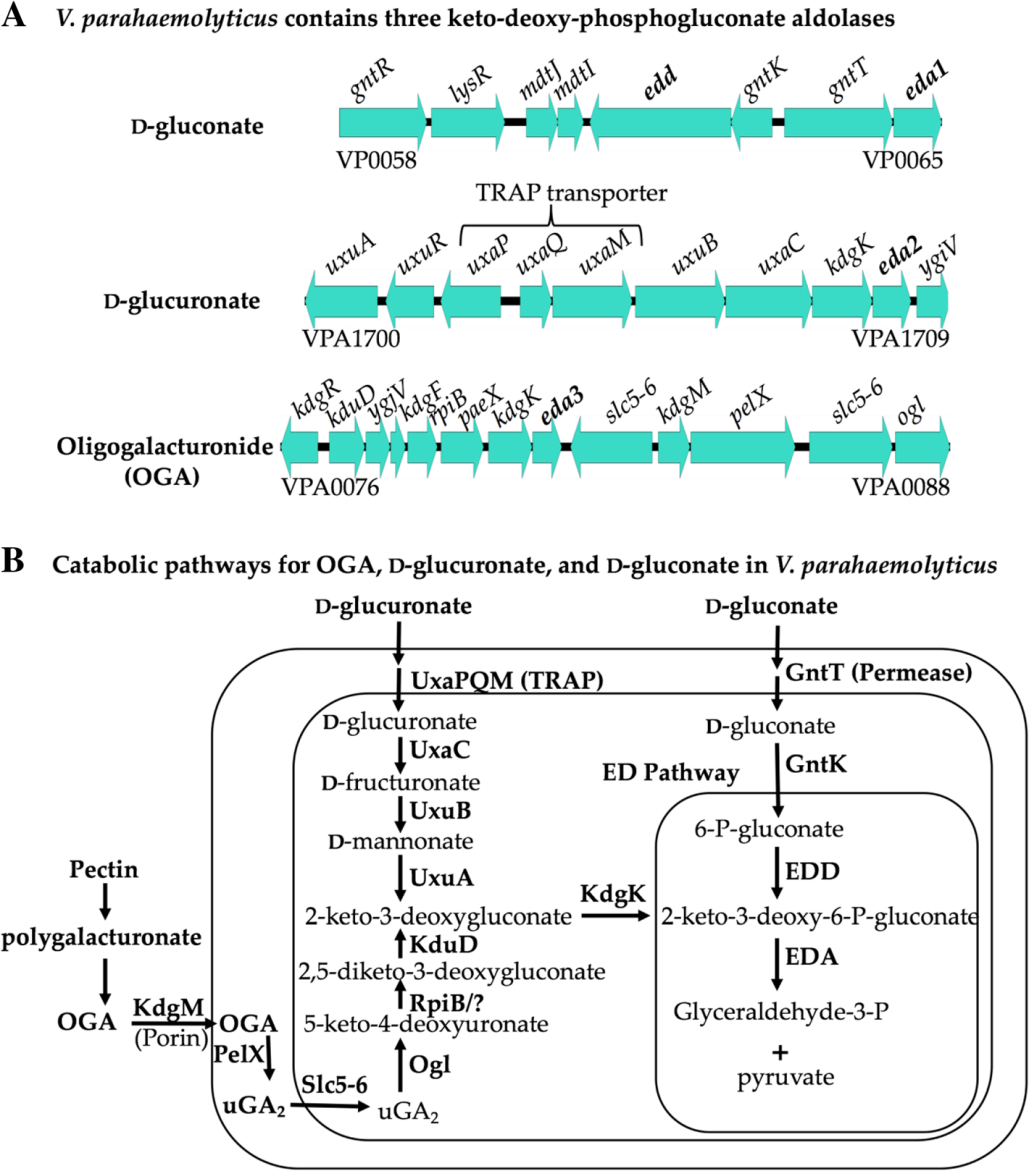
Three keto-deoxy-phosphogluconate aldolases (EDA1, EDA2, and EDA3). **a**. Gene cluster of d-gluconate, d-glucuronate and oligogalacturonide (OGA) present in *V. parahaemolyticus* RIMD2210633. **b**. Predicted catabolic pathway for the utilization of OGA, d-glucuronate and d-gluconate based on the ORFs present in *V. parahaemolyticus* RIMD2210633

**Fig. 4:**
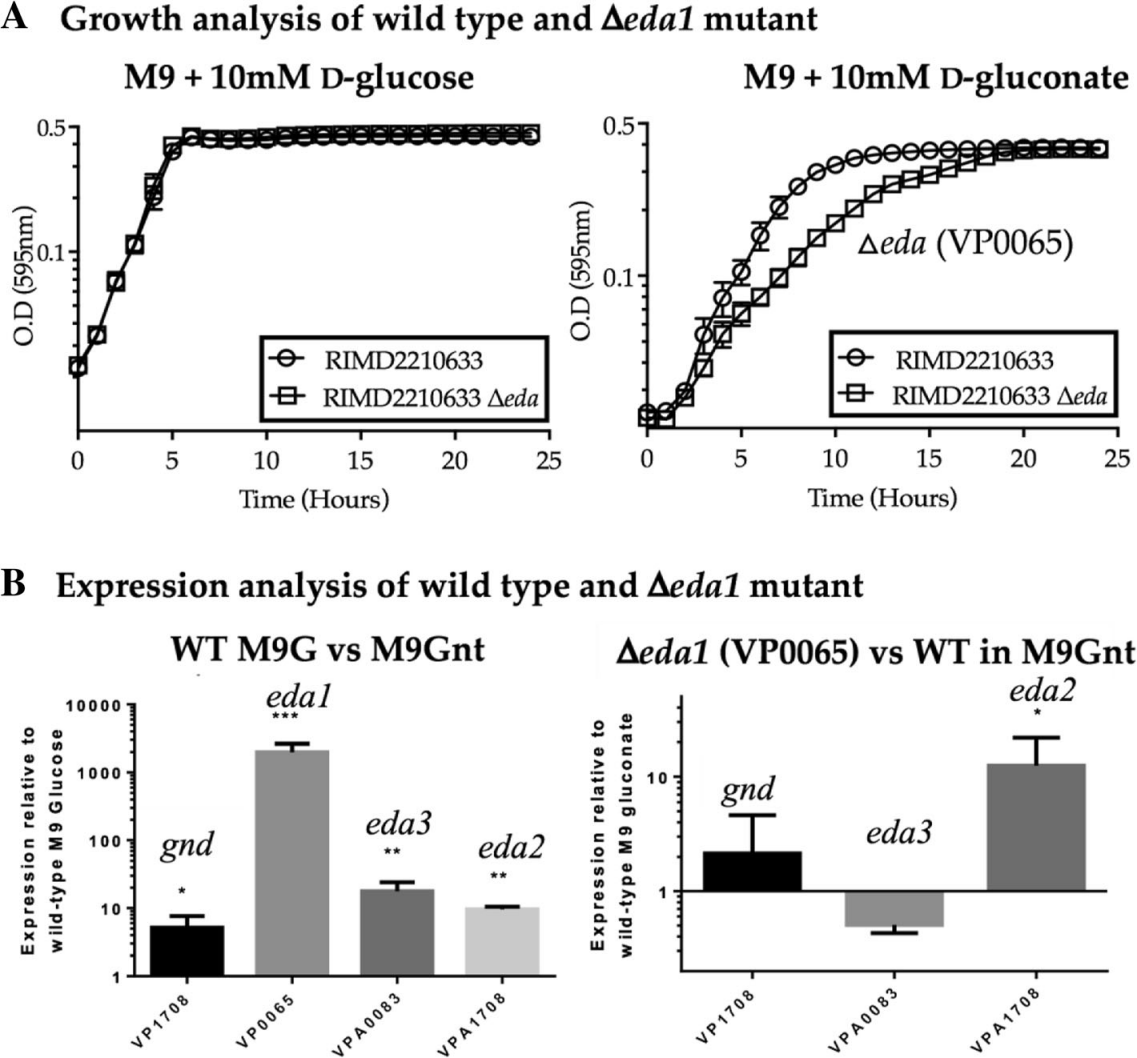
d-gluoconate metabolism. **a**. Growth analysis of *V. parahaemolyticus* RIMD2210633 and Δ*eda1* mutant strain in M9 supplemented with 10 mM D-glucose and 10 mM D-gluconate. **b**. Expression analysis of the first gene in the pentose phosphate pathway (VP1708) and the three putative aldolases (VP0065, VPA0083, VPA1708) in wild-type strain in gluconate relative to the expression of these genes in wild-type in glucose. Expression analysis of VP1708 and the two putative aldolases (VPA0083, VPA1708) in the Δ*eda1* mutant strain in gluconate relative to the expression of these genes in the wild-type in gluconate. *p < 0.05, **p < 0.01, ***p < 0.001

Entner-Doudoroff (ED) pathway, an alternative to the Embden-Meyerhof-Parnas (EMP) glycolytic pathway, utilizes two major enzymes, 6-phosphogluconate dehydratase (EDD) and 2-keto-3-deoxy-6-phosphogluconate (KDPG) aldolase (EDA) to convert glucose into pyruvate and glyceraldehyde-3-P [30]**.** An intermediate of this pathway, 6-phosphogluconate, can also be generated after phosphorylation of d-gluconate by gluconokinase (*gntK*) upon uptake. d-gluconate is a six-carbon acid, which is one step more oxidized than glucose and is present in a wide variety of environments for use as a carbon and energy source for many bacterial species. Enteric bacteria typically use the ED pathway for the inducible metabolism of d-gluconate. EDA is involved in d-glucuronate, d-galactonate, gluconides, and possibly glyoxylate metabolism, some of whose metabolic pathways were previously identified in *V. parahaemolyticus* (Fig. 3a and b) [31]. Both RIMD2210633 and UCM-V493 grew on d-gluconate as a sole carbon source, although similar to other sugars, UCM-V493 grew to a higher OD (Additional file 1. Table S1). In *V. parahaemolyticus*, the predicted gluconate catabolism genes are clustered together within chromosome 1 with *gntK-edd* divergently transcribed from *gntT-eda*, whereas in *E. coli* these genes are more dispersed in the chromosome (Fig. 3a)*.* In *V. cholerae*, the ED pathway is an essential requirement for d-gluconate catabolism [32]. We constructed an in-frame deletion of *eda* (VP0065) and examined growth in M9 minimal media supplemented with glucose (M9G) and M9 supplemented with gluconate (M9Gnt). Both mutant and wild type strains showed identical growth patterns when grown on M9G (Fig. 4a). Surprisingly, the *eda* (VP0065) mutant was still capable of growing in M9Gnt as the sole carbon source (Fig. 4a). Using VP0065 as a seed, BLAST analysis identified two additional EDA homologues within *V. parahaemolyticus* RIMD2210633. VPA0083 (EDA3) and VPA1708 (EDA*2*) showed 78 and 60% amino acid identity to VP0065 (EDA1), respectively. VPA0083 was within a cluster for oligogalacturonide (OGA) metabolism and VPA1708 was present within a cluster for the metabolism of d-glucuronate (Fig. 3a and b). RIMD2210633 grew on D-glucuronate as a sole carbon source but UCM-V493 did not (Additional file 1: Table S1). The d-glucuronate gene cluster is absent from UCM-V493.

The *eda1* mutant compared to wild type had a longer doubling time but reached a similar final biomass to wild type, which suggested that the *eda1* mutant may have been complemented by either *eda2* or *eda3* (Fig. 4a). Expression analysis in M9G compared to M9Gnt showed that all three *eda* genes were induced, with *eda1* showing the highest expression levels in M9Gnt (Fig. 4b). We also examined *gnd,* which encodes phosphogluconate dehydrogenase, a key enzyme in the Pentose Phosphate (PP), which can serve as an alternative pathway in gluconate catabolism in some species. The expression data showed a slight induction of *gnd* (VP1708), which suggests this pathway is likely not a major player in gluconate catabolism but does suggest the pathway is functional (Fig. 4b). Next, we examined expression of *eda2, eda3* and *gnd* in the Δ*eda1* mutant compared to wild type in M9Gnt and found that only *eda2* (VPA1708) was significantly induced (Fig. 4b). This suggested that *eda2* may be compensating for the lack of *eda1* when grown on d-gluconate as a sole carbon source.

**Fig. 5:**
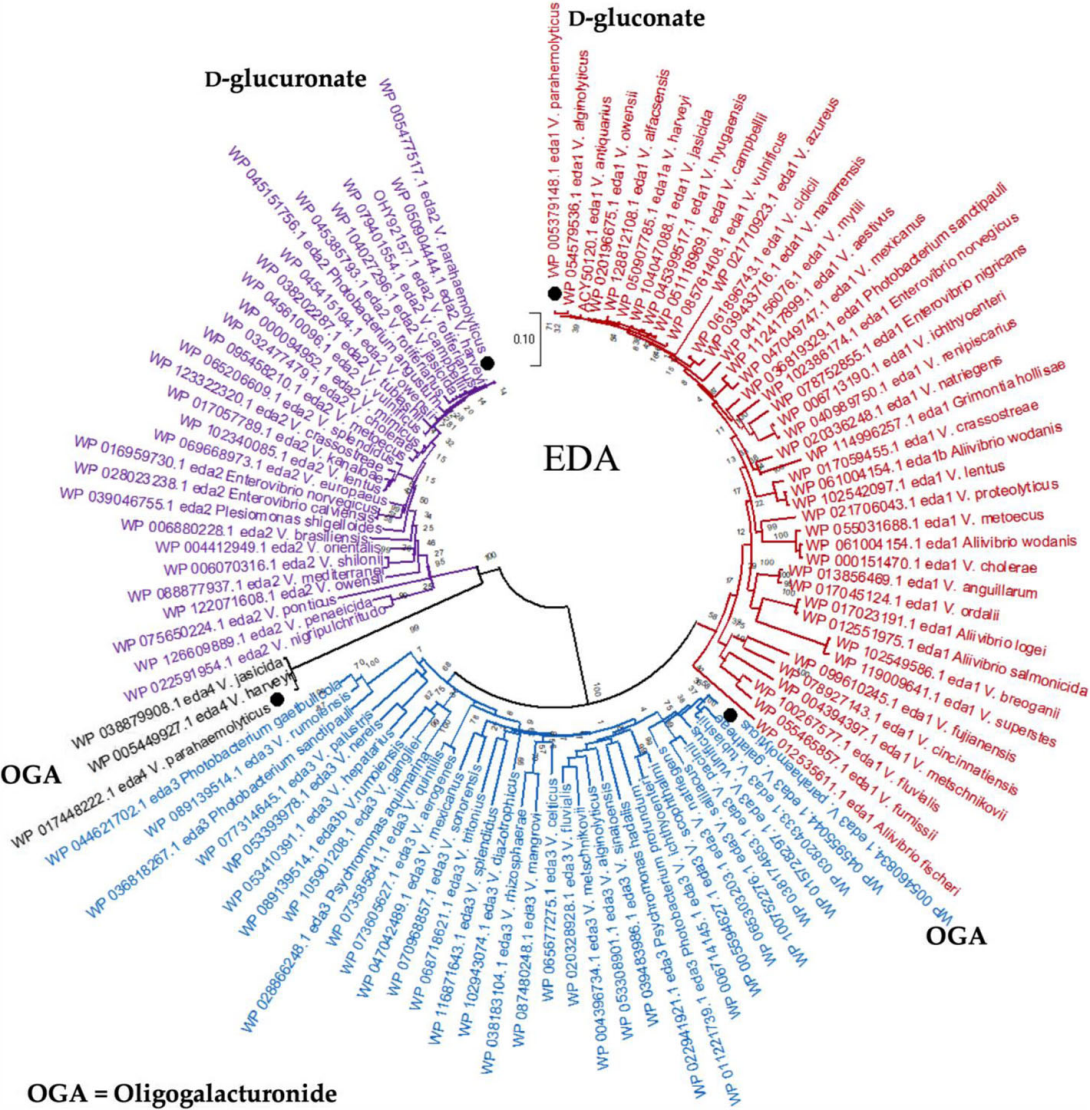
Phylogenetic analysis of EDA1, EDA2, EDA3 and EDA4. This analysis involved 108 amino acid sequences representing EDA1, EDA2, EDA3 and EDA4 homologues present in the *Vibrionaceae*. The evolutionary history was inferred using the Neighbor-Joining method [26]. The optimal tree with the sum of branch length = 6.21447650 is shown. The percentage of replicate trees in which the associated taxa clustered together in the bootstrap test (1000 replicates) are shown next to the branches [27]. The tree is drawn to scale, with branch lengths in the same units as those of the evolutionary distances used to infer the phylogenetic tree. The evolutionary distances were computed using the Dayhoff matrix based method [28] and are in the units of the number of amino acid substitutions per site. The rate variation among sites was modeled with a gamma distribution (shape parameter = 5). All ambiguous positions were removed for each sequence pair (pairwise deletion option). There were a total of 213 positions in the final dataset. Evolutionary analyses were conducted in MEGA X [29]

Bioinformatics analysis revealed that EDA1 (VP0065) is present in all *V. parahaemolyticus* and many members of the Campbellii clade, in general. In order to determine the evolutionary relationships and history of EDA, we reconstructed the phylogeny of EDA homologues present in the *Vibrionaceae* family (Fig. 5). This analysis indicated that EDA1 from members of the Campbellii clade are all closely related clustering together on the tree with short branch lengths (Fig. 5). Clustering with this group is the EDA1 protein from *V. vulnificus*, a species that is distantly related to the Campbellii clade, indicating that EDA1 has a shared evolutionary history in these species (Fig. 5). EDA1 is also present in most *V. cholerae* strains and clustered with EDA1 from *V. metoecus*, a close relative of this species. Within one of the most divergent branches of the EDA tree are proteins from *V. fluvalis* and *V. furnissii,* which are member of the Cholerae clade. This suggest the EDA1 was acquired independently within these closely related species, although alternatively in *V. fluvalis* and *V. furnissii*, EDA1 could be evolving faster. Overall, phylogenetic analysis suggests that EDA1 is phylogenetically widespread in the *Vibrionaceae* but is only present in a small subset of members of the family.

EDA2 was present within the d-glucuronate metabolism cluster in *V. parahaemolyticus* (Fig. 3a). Similar to EDA1, EDA2 is prevalent among the Campbellii clade members, however it is not always present in all members of a species (Fig. 5). For example, about half the *V. parahaemolyticus* genomes in the database contained EDA2. EDA2 was present in a handful of *V. cholerae* strains and the phylogeny of EDA2 suggests it was lost from *V. cholerae* given it is present in the closely related species *V. metoecus, V. mimicus,* and *V. fluvalis,* which all cluster together and most representatives contained the protein. EDA2 had a limited distribution among *Vibrionaceae* in general and was distantly related to EDA1. EDA3 is associated with an OGA metabolism cluster, which is involved in pectin metabolism and part of the KdgR regulon in *V. parahaemolyticus* [33]. EDA3 is more closely related to EDA1 than EDA2. EDA3 is present in all strains of *V. parahaemolyticus* and in all strains of *V. alginolyticus,* but these closely related species are present on divergent branches on the EDA3 tree. EDA3 is present in all *V. vulnificus* isolates and clustered closely with EDA3 from *V. parahaemolyticus* with no other major representatives of the Campbellii clade containing this protein (Fig. 5).

### Whole genome comparative analysis

The differences amongst strains in the presence of hexuronate catabolic clusters led us first to perform whole genome comparative analysis among 17 *V. parahaemolyticus* completed genomes (Additional file 1: Table S3). These 17 strains represented clinical strains that contain T3SS-2 and environmental strains. These strains were recovered from a range of different sources between 1951 and 2015 from Asia, Europe, South and North America (Additional file 1: Table S3). A summary of the regions of difference is found in (Additional file 1: Table S4). We identified several distinct metabolic regions that were variably present among the strains. In particular, a metabolic island was present within chromosome 2 that contained a citrate fermentation cluster, an l-rhamnose cluster, and a second OGA metabolism cluster that also contained a KDPG aldolase that we named EDA4. A second region was identified that contained a non-homologous L-rhamnose metabolism cluster. A limited number of strains contained an island with an L-arabinogalactan metabolism cluster.

### A metabolic island that harbors a citrate fermentation gene cluster

**Fig. 6:**
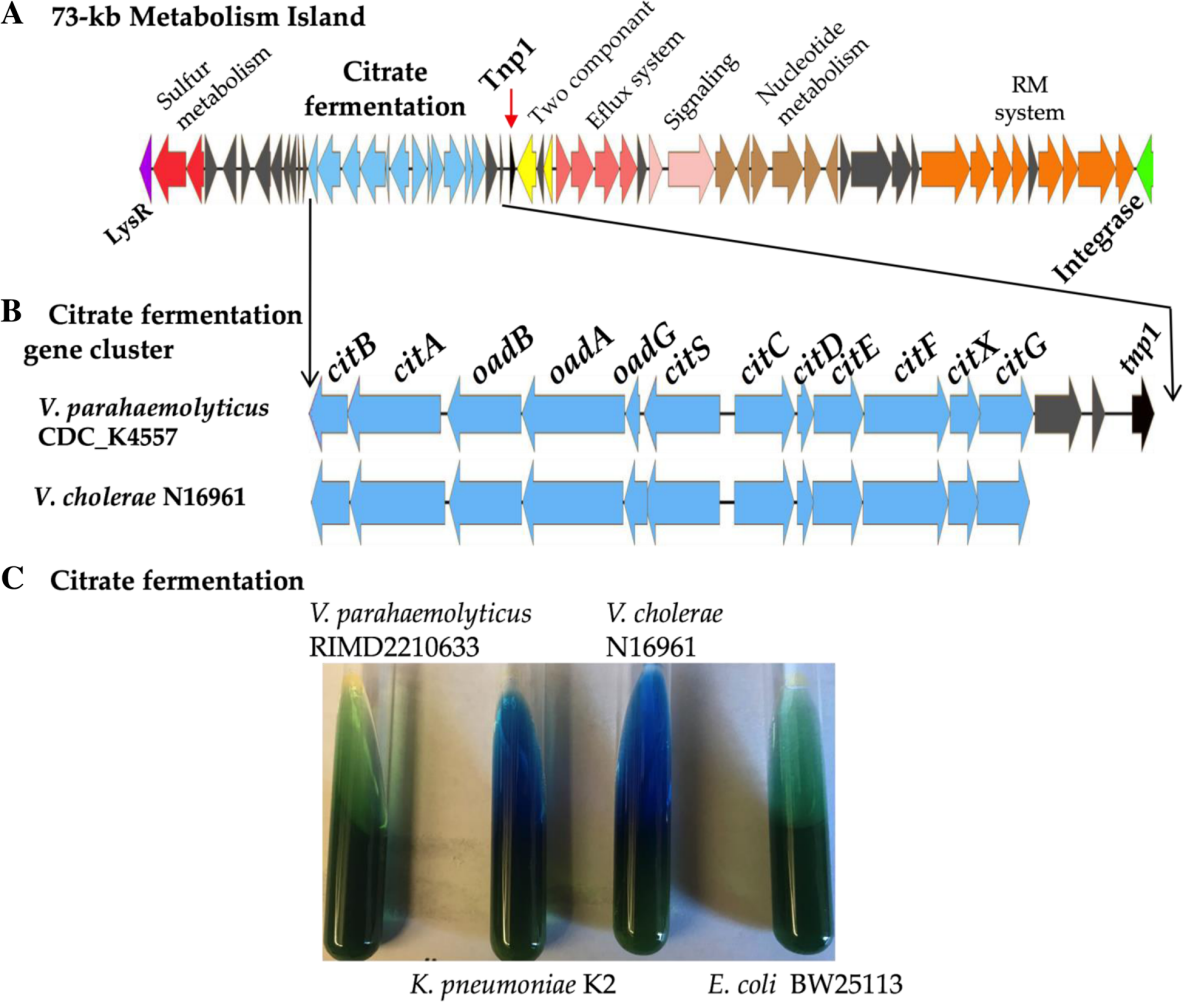
A 73-kb metabolic island in *V. parahaemolyticus*. **a**. Detailed analysis of 73-kb metabolic island identified in chromosome 2 of CDC_K4557, which was missing from RIMD2210633. Arrows indicate ORFs and ORFs with identical color indicate similar function. Gray arrows, genes coding hypothetical and other functional proteins. Black arrow represents a transposase (Tnp). **b**. Detailed analysis of the citrate fermentation cluster in CDC_K4557 and comparison of the same region in *V. cholerae*. **c**. Citrate fermentation in Simmons citrate slant. Conversion of green slant to blue indicates citrate utilization

Comparative genome analysis of RIMD2210633 and CDC_K4557 identified a 73-kb genomic island on chromosome 2 between VPA0712 and VPA0713 relative to RIMD2210633, which lacked the region. The metabolic island showed all the features of a horizontally acquired region; it was flanked by an integrase at the 3′ end, contained attachment sites attL and attR, which indicated site specific integration, and had a %GC content of 42.6% lower than 45.35% of the entire genome (Fig. 6a) [34–38]. The island contained a complete citrate fermentation system (Fig. 6b). In *V. cholerae* citrate fermentation is a hallmark physiological test for its identification and the genes required are present in all strains of the species. In *V. parahaemolyticus* RIMD2210633, only homologues of *oadA oadB oadG (*VP2543-VP2545) are present and it cannot grow on citrate anaerobically (Fig. 6c). In *V. parahaemolyticus* CDC_K4557, the citrate fermentation cluster was similar to the cluster present in *V. cholerae,* and adjacent to the cluster was a transposase (Fig. 6b). BLAST analysis showed that the citrate fermentation cluster was present in a total of 39 *V. parahaemolyticus* strains within the same island (Additional file 1: Table S5). Of the 39 strains, 34 were pathogenic and harbored a T3SS-2α system. This island was present in clinical isolates recovered in the 1980s, 1990s and 2000s. Interestingly, all 20 isolates in the database that were isolated during the *V. parahaemolyticus* outbreak in Peru in 2009 contained this region (Additional file 1: Table S5). These *V. parahaemolyticus* outbreak strains belonged to ST 120 clonal group that originated in China [39]. We speculate that the ability to utilize citrate, in part, may have provided the Peru clone with a competitive advantage, during the epidemic in 2009.

**Fig. 7:**
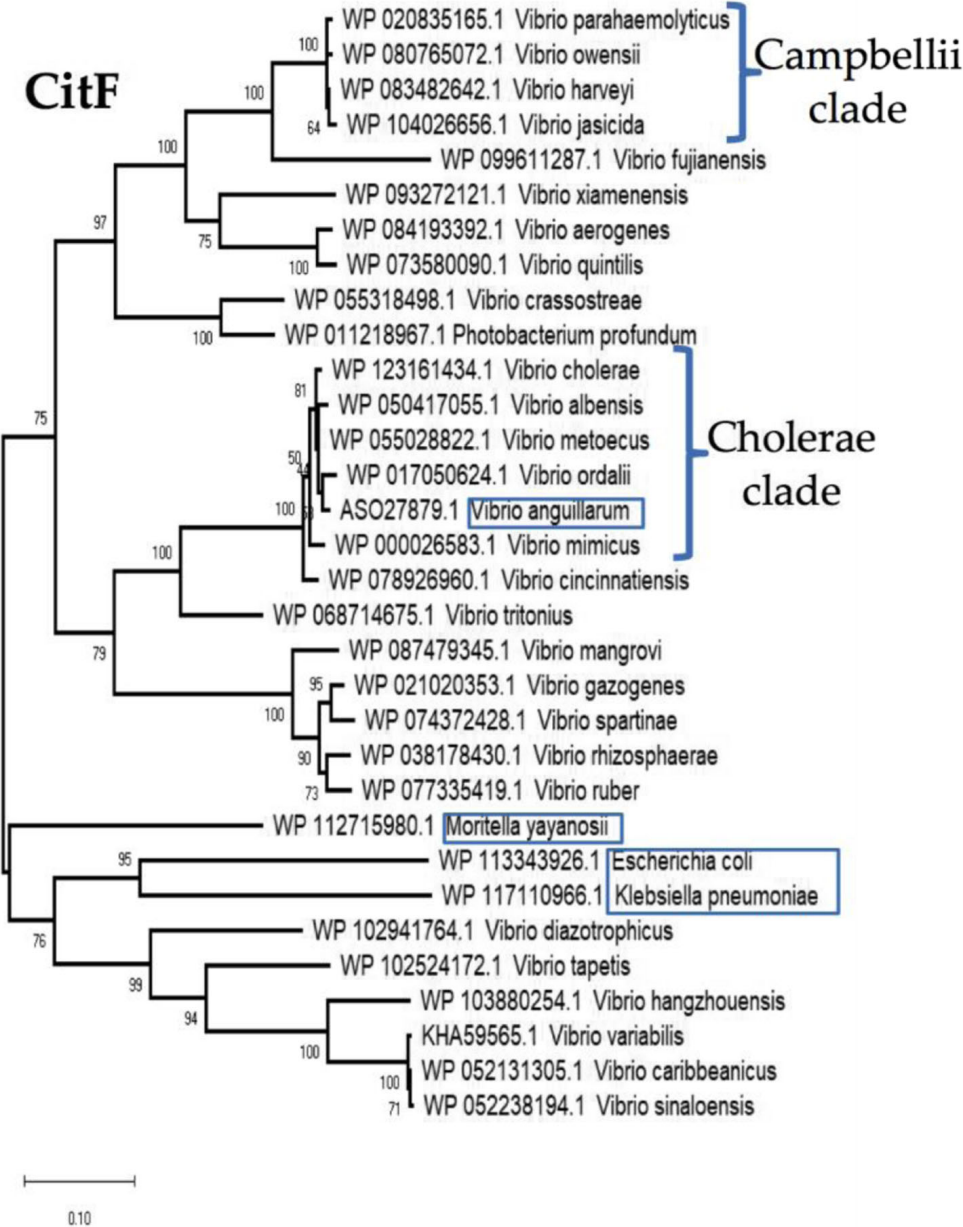
Phylogenetic analysis of CitF among the *Vibrionaceae*. This analysis involved 32 amino acid sequences representing CitF homologues present among the *Vibrionaceae*. Phylogeny was inferred by using the Maximum Likelihood method and Le_Gascuel_2008 model [40]. The tree with the highest log likelihood (− 7580.58) is shown. The percentage of trees in which the associated taxa clustered together is shown next to the branches. Initial tree(s) for the heuristic search were obtained automatically by applying Neighbor-Join and BioNJ algorithms to a matrix of pairwise distances estimated using a JTT model, and then selecting the topology with superior log likelihood value. A discrete Gamma distribution was used to model evolutionary rate differences among sites (5 categories (+*G*, parameter = 0.4398)). The tree is drawn to scale, with branch lengths measured in the number of substitutions per site. All positions with less than 95% site coverage were eliminated, i.e., fewer than 5% alignment gaps, missing data, and ambiguous bases were allowed at any position (partial deletion option). There were a total of 489 positions in the final dataset. Evolutionary analyses were conducted in MEGA X [29]

To examine the evolutionary history of citrate fermentation within *V. parahaemolyticus* and among the *Vibrionaceae*, we performed a phylogenetic analysis of representative CitF proteins from this group (Fig. 7). The limited distribution of CitF within *V. parahaemolyticus* (> 5% of sequenced strains) indicates that it is a recent addition to the species and was likely acquired from a closely related species. Within the Cholerae clade (*V. cholerae, V. metoecus, V. mimicus, V. albensis*), CitF is present in all members of the species and these species have closely related CitF proteins indicating that the protein maybe ancestral to the group. Interestingly within this group is CitF from *V. anguillarum* strains, a species that is distantly related to this clade. This suggest that *V. anguillarum* acquired CitF by horizontal gene transfer between these species. The CitF distribution was confined to a limited number of *Vibrionaceae* species that formed highly divergent branches with several CitF proteins from *Vibrio* species clustering with CitF from *E. coli* and *Klebsiella pneumonia* (Fig. 7).

Variants of the 73-kb genomic island containing the citrate fermentation cluster were identified (Additional file 2: Figure S1). In strain CDC_K5635, the metabolic island contained a CRISPR-Cas system. Our analysis identified the CRISPR-Cas system as a type I-F system, which contained the *cas* operon of *cas1cas2/3cas8cas5cas7cas6f*, a leader sequence and a CRISPR array. The CRISPR array contained a typical type I-F repeat and 34 spacers, which suggested the system was functional. The CRISPR-Cas system was inserted adjacent the citrate gene cluster (Additional file 2: Figure S1). In strain 3259, the metabolic island was 105-kb and in addition to the citrate cluster and CRISPR-Cas system also contained the genes for l-rhamnose and OGA catabolism (Additional file 2: Figure S1). Associated with each of these function modules were transposases suggestion a possible mechanism of acquisition within the island (Additional file 2: Figure S1). A previous study identified this type I-F CRISPR-Cas system in a total of 10 *V. parahaemolyticus* strains, all recovered in the mid-2000s [17].

### l-rhamnose utilization clusters within two different genomic islands

**Fig. 8:**
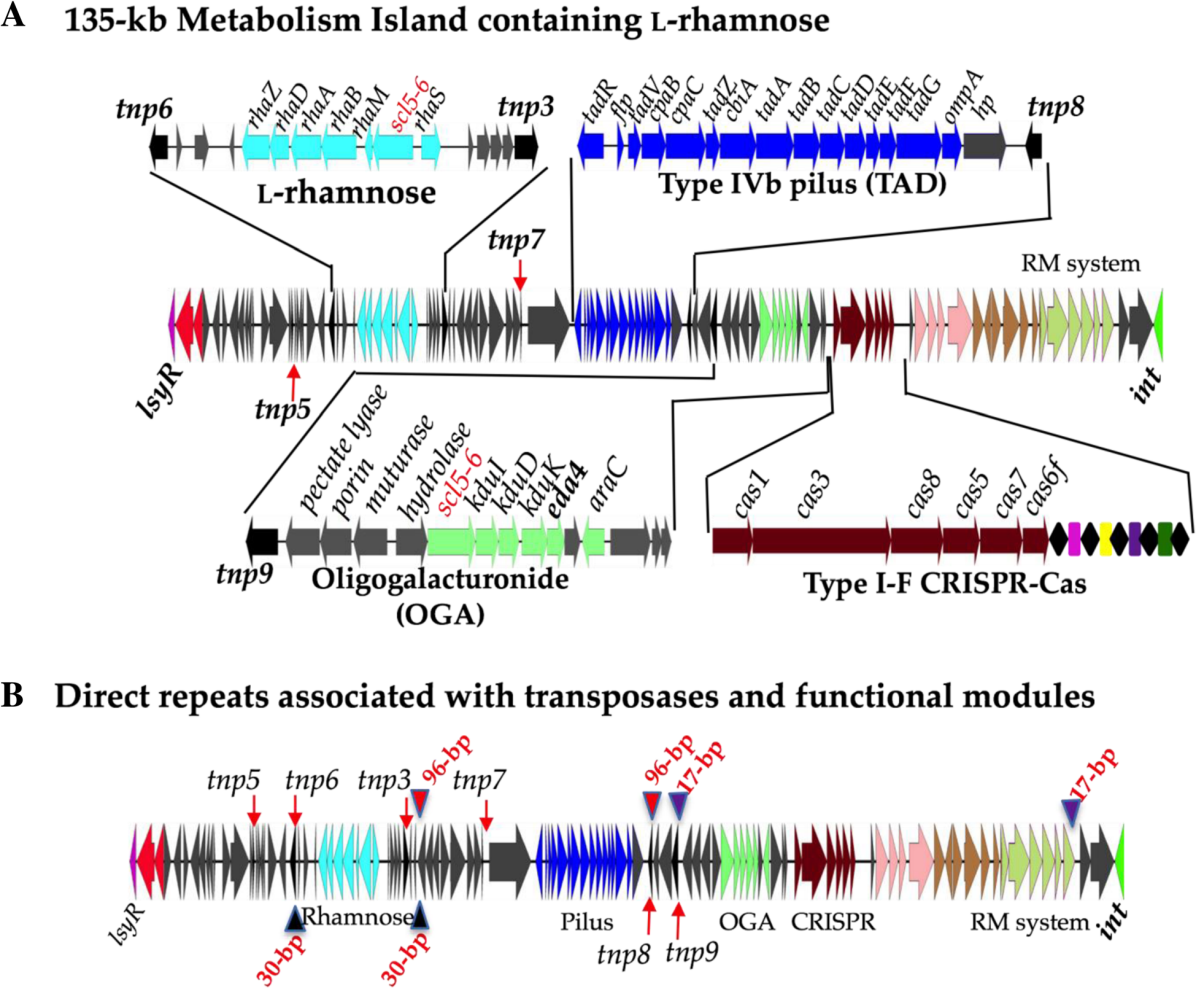
A 135-kb Metabolism island in *V. parahaemolyticus*. **a**. Detailed analysis of a 135-kb metabolic island identified in chromosome 2 of FORC_022 absent from RIMD2210633. Arrows indicate ORFs and ORFs with identical color indicate similar function. Gray arrows, genes coding hypothetical and other functional proteins. Black arrows represent transposases. **b**. Transposases and direct repeats identified flanking the functional modules with the 135-kb island

l-rhamnose, a deoxy-hexose, is commonly found in plants and bacterial cell walls [41]. Genome comparative analysis of RIMD2210633 and FORC_022, identified a 135-kb island related to the 73-kb metabolic island described above but lacked the citrate fermentation gene cluster (Fig. 8a). Similar to strain 3259, in FORC_022, the metabolic island contained homologues of *rhaM, rhaA, rhaB, rhaD,* a SCL5–6-like sodium symporter, *rhaZ,* and a regulator encoded by *rhaS,* all required for l-rhamnose utilization (Fig. 8a). In addition, the island contained a type IV pilus gene cluster and an oligogalacturonide (OGA) catabolism cluster (Fig. 8a). The OGA cluster contained homologues of *kduI, kduD, kdgK,* and another KDPG aldolase EDA4. The EDA4 protein shared 50% amino acid identity with EDA3 (VPA0083), 53% with EDA1 (VP0065) and 57% identity with EDA2 (VPA1708). The distribution of EDA4 was limited, present in some *V. harveyi* and *V. jasicida* strains (Fig. 5). Within the OGA cluster, a SCL5–6-like sodium symporter, an AraC family regulator, two hydrolases, and a porin required for the uptake and catabolism of OGA and unsaturated monomers of pectin were identified (Additional file 2: Figure S2B). Within this island a complete type I-F CRISPR-Cas system was present similar to the system present in strains CDC_K5635 and 3259 but with different sized CRISPR arrays and spacer sequences in each strain. Strain 3259 contained 4 spacers, FORC_022 had 28 spacers and CDC_K5635 had 34 spacers suggesting these systems were active and acquiring spacers.

BLAST analysis identified an additional 19 strains that harbored a homologous l-rhamnose cluster associated with variants of this island, at the same genomic location on chromosome 2 (Additional file 1: Table S6). These strains were predominantly isolated from Asia from 2006 to 2013 and most strains were non-pathogenic (lacked a T3SS-2 system) (Additional file 1: Table S6). This suggests that the L-rhamnose cluster was recently acquired in a subpopulation of bacteria. In strain RM-13-3, a 119-kb island similar to the 135-kb metabolic island was present but lacked the OAG and CRISPR-Cas clusters (Additional file 2: Figure S2A). In strain TUMSAT_H10_H6, a 74-kb variant of the metabolic island was present that contained only the l-rhamnose and CRISPR-Cas clusters (Additional file 2: Figure S2A). In TUMSAT_H10_H6, the type I-F CRISPR array contained 24 spacers, which did not share homology with spacer sequences from other type I-F systems identified in *V. parahaemolyticus*. The type IV pilus cluster, OGA cluster, and the l-rhamnose clusters all had transposase genes nearby and we identified direct repeats flanking these functional modules (Fig. 8b). This suggested to us a mechanism of acquisition as discrete modules within the island by a transposon like mechanism. Overall, these data indicate that this metabolic island is a dynamic region with the loss and gain of functional modules occurring over evolutionarily short timescales and confined to subpopulations of bacteria.

**Fig. 9:**
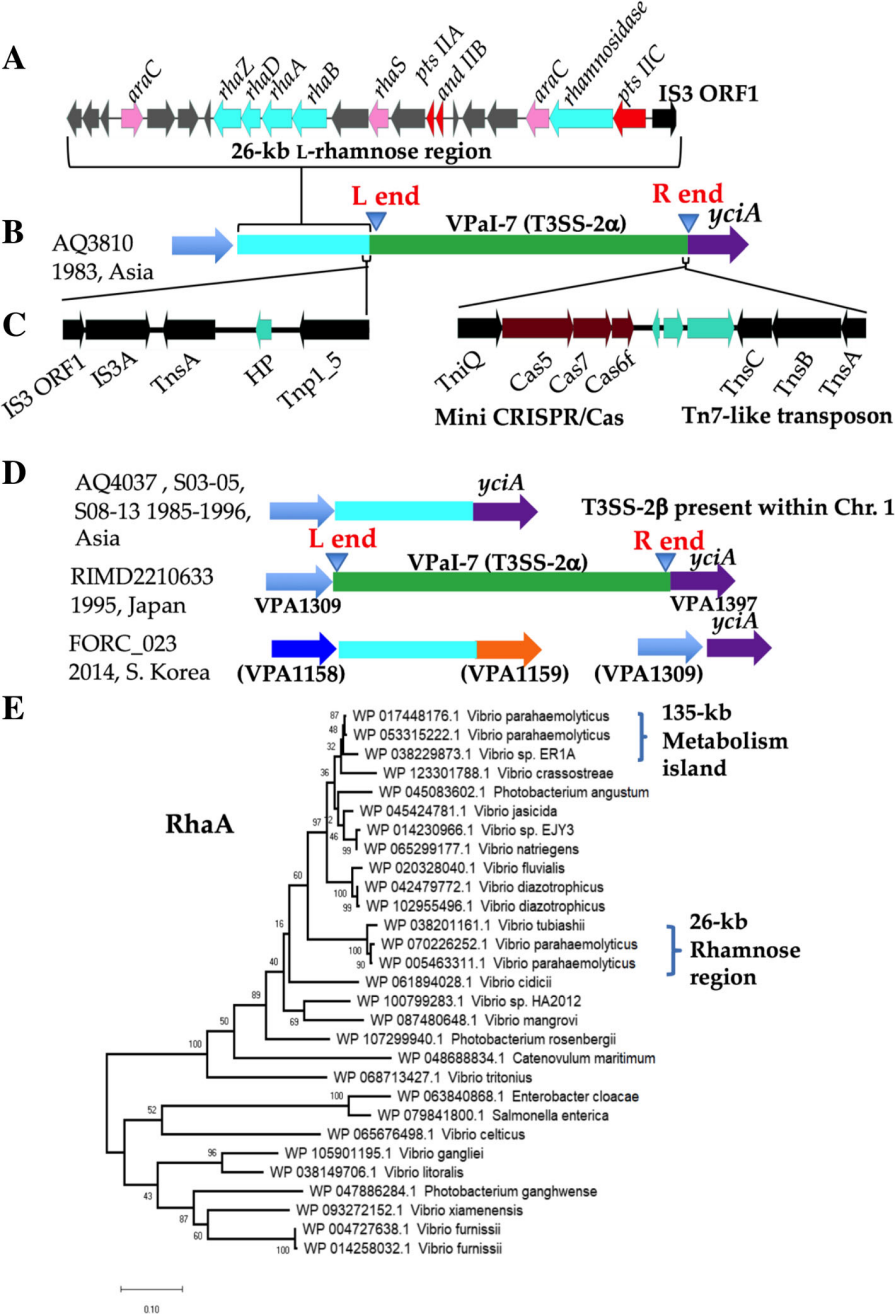
Analysis of the 26-kb l-rhamnose utilization island. **a**. Gene cluster of the l-rhamnose utilization in *V. parahaemolyticus* AQ3810. **b**. Genomic locus of the 26-kb l-rhamnose region in AQ3810. **c**. A Tn7-like transposon and a mini CRISPR-Cas system associated with T3SS-2α and a transposon like region associated with the 26-kb L-rhamnose region. **d**. Schematic of the L- rhamnose genomic loci in AQ4037, S03-S05, S08-S13 VPA1309 in chromosome 2 that is also the location of T3SS-2α in RIMD2210633. Location of the 26-kb l-rhamnose island in FORC_023 and the empty site at VPA1309. Parenthesis indicates homologous ORFs. **e**. Phylogenetic analysis of RhaA protein from FORC_022 and AQ3810 among members of the family *Vibrionaceae*. The evolutionary history was inferred by using the Maximum Likelihood method and Le_Gascuel_2008 model [40]. The tree with the highest log likelihood (− 6340.56) is shown. The percentage of trees in which the associated taxa clustered together is shown next to the branches. Initial tree(s) for the heuristic search were obtained automatically by applying Neighbor-Join and BioNJ algorithms to a matrix of pairwise distances estimated using a JTT model, and then selecting the topology with superior log likelihood value. A discrete Gamma distribution was used to model evolutionary rate differences among sites (5 categories (+*G*, parameter = 0.4168)). The tree is drawn to scale, with branch lengths measured in the number of substitutions per site. This analysis involved 29 amino acid sequences. All positions with less than 95% site coverage were eliminated, i.e., fewer than 5% alignment gaps, missing data, and ambiguous bases were allowed at any position (partial deletion option). There were a total of 418 positions in the final dataset. Evolutionary analyses were conducted in MEGA X [29]

Interestingly, we identified 12 strains with a second l-rhamnose cluster, which shared only 74 to 86% amino acid identity with the cluster carried within the metabolic island (Additional file 1: Table S6 and Fig. 9a). In strain AQ3810, the l-rhamnose cluster was present within a 26-kb region that was inserted at ORF VPA1309 relative to RIMD2210633, which lacked the region. Adjacent to the 26-kb region were several transposase genes. Both strains contain the T3SS-2α region at this site (Fig. 9b and d). The 26-kb l-rhamnose region contained a gene encoding rhamnosidase suggesting an ability to catabolize l-rhamnose oligosaccharides. No canonical l-rhamnose or l-rhamnose oligosaccharide transporter was identified but a PTS system was adjacent to the l-rhamnose catabolic genes (Fig. 9a). In strains that have a T3SS-2β present on chromosome 1, the 26-kb L-rhamnose region is at VPA1309 with the same transposases as described above (Fig. 9d and Additional file 1: Table S6). In FORC_023 isolated in South Korea in 2014, the 26-kb region was present between VPA1158 and VPA1159 relative to RIMD2210633 (Fig. 9d). Thus, our genomic data shows that *V. parahaemolyticus* acquired the l-rhamnose catabolic cluster at least three times during its evolution, perhaps suggesting an important fitness phenotype in certain niches.

The phylogeny of RhaA showed two divergent branching patterns representing RhaA from the 135-kb metabolic island and the 26-kb region. RhaA from the metabolic island is present in several species belonging to the Campbellii clade, and are closely related, clustering together with short branch length **(**Fig. 9e). The RhaA from the 26-kb region forms a divergent branch related to RhaA in other Campbellii clade species. Highly divergent RhaA proteins were present in several *Vibrio* species that were more closely related to RhaA from *Salmonella enterica* and *Enterobacter cloacae* than to other *Vibrio* species (Fig. 9e). Overall, the data suggest that RhaA is not phylogenetically widespread and was acquired multiple times within the *Vibrionaceae*.

### T3SS-2 present within a Tn7-like transposon-CRISPR-Cas element

In strain AQ3810, adjacent to the 26-kb l-rhamnose region was the VPaI-7 region that contains T3SS-2α (Fig. 9b and c). In this strain, the VPaI-7 region is flanked by Tn7-like transposon right end and left end attachment sites (named L end and R end) (Fig. 9b and d). The R end and L end sequences are absent from strains that do not have a T3SS-2 at this site (Fig. 9d). Adjacent to the R end is a three gene cluster that showed homology to Tn7 transposon genes *tnsABC*. The Tn7 genes *tnsD* and *tnsE*, involved in target site insertion, are absent but a homologue *tnsD* homologue *tniQ* is present within a mini CRISPR-Cas system (*tniQcas5cas7cas6*) close to the Tn7 gene cluster (Fig. 9c). In all *V. parahaemolyticus* strains that contained a T3SS-2α system in chromosome 2 that we examined, the mini CRISPR-Cas system and the Tn7-like region always co-occurred with T3SS-2α. The mini CRISPR-Cas system depending on the strain has either no array or a very short array consisting of one or two spacers suggesting it is unable to acquired new spacers since it lacks Cas1 and Cas2. The juxtaposition of a Tn7-like transposon and a mini-CRISPR-Cs system has previously been noted in several *Vibrio* species [17]. It has been proposed that the Tn7-like transposon and mini CRISPR-Cas function together to form a function transposon that allows for target site integration [17, 42, 43]. This suggests a novel non-canonical function for a CRISPR-Cas system; co-opted by a transposon for the mobilization of bacterial DNA.

### l-arabinogalactan catabolism gene cluster within a novel metabolic island

l-arabinose is a pentose sugar and is an important nutrient source in vivo as it is one of the sugars found in the proteoglycan of the mucin present in the gastrointestinal track [21]. An l-arabinose catabolic cluster is present in *V. parahaemolyticus* RIMD2210633 and this strain can grow on l-arabinose as a sole carbon source **(**Additional file 1: Table S1) [20]. UCM-V493 cannot grow on l-arabinose as a sole carbon source and lacks the gene cluster required **(**Additional file 1: Table S1). The catabolic and ABC-type transporter genes for l-arabinose utilization are present within a 9.5-kb region present in most pathogenic *V. parahaemolyticus* strains in the database (Additional file 2: Figure S3C). A notable exception is the absent of this cluster from strains that contained the T3SS-2γ system (Additional file 1: Table S2). Bioinformatics analysis identified in FORC_008, a second l-arabinose cluster that only shared ~ 70% amino acid identity with that from RIMD2010633 and contained an uncharacterized major facilitator superfamily (MFS) type transporter (Additional file 2: Figure S3A). This cluster was carried within a 13.2-kb region inserted between VPA0441 and VPA0449 with respect to RIMD2210633 (Additional file 2: Figure S3A). The region was present in 21 non-pathogenic strains, 15 pathogenic strains that contained T3SS-2β, and one pathogenic strain with T3SS-2α (Additional file 1: Table S7). This region also contained α-l- and β-l-arabinofuranosidases, an uncharacterized hydrolase and a second MFS type transporter suggesting an ability to catabolize l-arabinose oligomers (Additional file 2: Figure S3A). The data indicate that *V. parahaemolyticus* has acquired two non-homologous l-arabinose utilization systems during its evolution. Interestingly, in RIMD2210633 between VPA0441 and VPA0449, a putative peptide modification system, His-Xaa-Ser, was present, whereas in strain CFSAN007457 a complete d-galactitol utilization system was present at the same position (Additional file 2: Figure S3A and S3B). The d-galactitol utilization system was identified in 15 strains, mostly North American isolates containing T3SS-2β isolated from 1984 to 2010 and a few non-pathogenic strains (Additional file 1: Table S7).

To determine the evolutionary history of the L-arabinose clusters in *V. parahaemolyticus* RIMD2210633 and FORC_008, we reconstructed a phylogenetic tree based on AraD proteins from both cluster and examined their evolutionary relationships in *Vibrio* species. AraD from RIMD2210633 formed a distinct divergent cluster from AraD present in FORC_008 (Additional file 2: Figure S4). AraD from RIMD2210633 had a limited distribution outside of *V. parahaemolyticus* with only two uncharacterized species containing a closely related protein. Branching divergently and distant from this cluster were AraD proteins from over a dozen *Vibrio* species each having long-branch lengths. AraD from FORC_008 formed a tight cluster with AraD from several *Vibrio* species and *Grimontia hollisae*. These AraD proteins formed the most divergent branch on the tree indicating that they are not related to AraD present in RIMD2210633 (Additional file 2: Figure S4).

Comparative genomic analysis of RIMD2210633 and CH25, a non-pathogenic strain isolated from seafood in China, identified an l-arabinogalactan catabolic cluster (Additional file 2: Figure S5A). The genes for the uptake of l-arabinogalactan and its conversion to l-arabinose and d-galactose were clustered with *galETK* required for d-galactose catabolism (Additional file 2: Figure S5A and S5B). The 13 gene region was present within a 62-kb island on chromosome 2 that was absent from RIMD2210633**.** An integrase was present at the 3′ end of the island and the % GC of the island was 41% compared to 45% of the entire genome indicating that this island was horizontally acquired (Additional file 2: Figure S5). A gene encoding a transposase was identified next to the l-arabinogalactan cluster and the island was identified in 10 additional *V. parahaemolyticus* strains. These strains were typically from seafood samples with three strains belonging to the ST631 with a T3SS-2γ.

## Discussion

A key mechanism in the evolution of bacteria is horizontal gene transfer and the acquisition of cluster of genes that encode new phenotypes. Many studies have shown the importance of the acquisition of virulence genes on mobile genetic elements, such as phage, plasmids and pathogenicity islands, in the emergence of new pathogens and the enhancement of established pathogens [35–37, 44–49]. In *V. parahaemolyticus*, its emergence as a human pathogen resulted from the acquisition of T3SS-2 that is absent from non-pathogenic strains [8, 9]. Several studies have demonstrated differences in genome content among *V. parahaemolyticus* strains with an emphasis on potential virulence factors and pathogen markers [9, 14, 50–52].

However, other factors can play a key role in the emergence of new strains in new environments such as metabolic traits. Metabolic capacity is an important fitness factor both within the natural environment and within host species allowing bacteria to use new nutrient sources and/or acquire and use nutrients more efficiently. Little is known about how metabolic differences emerge or how widespread these differences are within and between species. This study examined both fine scale differences (transporter differences, multiple copies of the same protein) and absolute differences (present and absent of metabolism clusters) among *V. parahaemolyticus* isolates. We show that differences in the types of transporters present within a catabolism gene cluster can lead to differences in utilization efficiencies. The significance of possessing high and low affinity transporters will depend to a large extend on the niche a particular strain is present in. We found that several EDA homologues were present in *V. parahaemolyticus* and was a feature of the Campbellii clade in general, suggesting that sugar acids are an important nutrient source for this group.

We identified multiple metabolism islands carrying different catabolic gene clusters (citrate, l-rhamnose, l-arabinose, l-arabinogalactan, d-galactitol, and OGA) present in a range of *V. parahaemolyticus* strains and diverse *Vibrio* species. Some metabolic regions showed a correlation with the emergence of *V. parahaemolyticus* pathogenic clones such as the present of citrate fermentation in strains from 2009 Peru outbreak. On the other hand, many of the metabolic differences did not correlate with pathogenic strains, indicating that environmental fitness is an essential driver of metabolic diversity. Many of the metabolic traits examined had a limited distribution outside of the Campbellii clade, and others, like L-arabinose utilization, had a limited distribution outside of *V. parahaemolyticus,* suggesting perhaps a defining physiological feature of the species. Metabolic traits such as citrate fermentation had a limited distribution within *V. parahaemolyticus* and the Campbellii clade, in general but were prevalent among members of the Cholerae clade, a key biochemical test for *V. cholerae*.

The identification of transposase genes associated with the metabolic systems present on islands suggested a mechanism by which these regions are lost and gained. For the 135-kb metabolic island that contained multiple metabolism cluster inserted at VPA0712 relative to RIMD2210633, we identified a number of variant islands (Additional file 2: Figure S6). The most evolutionary parsimonious scenario for how these variants arose is that a progenitor island contained all three metabolic gene clusters, the type IV pilus and the type I-F CRISPR-Cas system (Additional file 2: Figure S6). We speculate that many of variant islands arose from single deletion events from this progenitor island whereas other variants arose by two deletion events (Additional file 2: Figure S6). The presence of transposase genes and direct repeats flanking the functional modules suggests a mechanism of how the progenitor island arose and how variant islands can be formed from deletion events. Of course, it is also possible that these variants could arise by accretions of systems within an island mediated by transposons as well but would require many more steps. The presence of these regions in both clinical and environmental strains indicates that the gene pool or reservoir for novel phenotypes is large.

The identification of a possible Tn7-like transposon-CRISPR-Cas system interaction involved in the mobilization of T3SS-2 systems within and between *V. parahaemolyticus* strains is curious and noteworthy. The T3SS-2 system genes are all within the Tn7-like transposon R end and L end attachment sites strongly suggesting the 80-kb region is carried on the transposon. The transposon genes *tnsABC* are adjacent to the insertion site *yciA*, which is typical of Tn7-like systems, but lack the *tnsD* or *tnsE* genes involved in target integration. A *tnsD* homologue, *tniQ* is present within a CRISPR-Cas operon *tniQcas5cas7cas6f*, which lacks the *cas1cas2* genes required for spacer acquisition. The mini CRISPR-Cas system always co-occurs with *tnsABC* not only in *V. parahaemolyticus* but also in other species with R end and L end sites [17, 43]. One could speculate that the mini CRISPR-Cas system allows the transposon to target and insert into novel sites permitting its propagation. Whether the two systems have co-opted each other to form a functional unit involved in the mobilization of large bacterial regions remains to be determined experimentally. Indeed, it will be an exciting prospect to determine whether this proposed non-canonical function of a CRISPR-Cas system plays a role in the acquisition of T3SSs.

Lastly, our phenotypic microarray analysis of growth of RIMD2210633 and UCM-V493 on different carbon sources demonstrate that UCM-V493 could use a wider array of carbon sources and use many substrates significantly better than RIMD2210633. Also, UCM-V493 could grow on tween 20, glycyl-L-glutamic acid, propionic acid and chondroitin sulfate c, whereas RIMD2210633 could not. Interestingly both strains grew significantly better on α and γ cyclodextrins than glucose. Whether these growth differences are strain specific or a reflection of wider patterns between clinical and environmental isolates within the species will need to be examined further.

## Conclusions

This study has identified many new metabolic features of *V. parahaemolyticus* that were previously unrecognized. Our study demonstrates that both catabolism gene clusters and single gene transporters can be acquired by horizontal transfer that can have significant effects on fitness. Phylogenetic analyses of these metabolism regions in many cases demonstrate independent evolution among *Vibrio* species suggesting convergent evolution of these phenotypic traits.

## Methods

### Bacterial strains, media, and culture conditions

All bacterial strains and plasmids used for functional analysis in this study are listed in Table 1. Bacterial strains used for in silico analysis are listed in Additional file 1: Table S3. Unless stated otherwise, all *V. parahaemolyticus* strains were grown in lysogeny broth (LB) medium (Fischer Scientific, Pittsburgh, PA) containing 3% NaCl at 37 °C with aeration or M9 medium media (Sigma Aldrich, St. Louis, MO) supplemented with 3% NaCl and carbon sources as required. For Simmons citrate test, bacterial colonies were inoculated by stabbing the Simmons citrate agar slant and the slant was incubated at 37 °C for 24 h. Antibiotics were added to growth media at the following concentration: streptomycin (Sm), 200 μg/ml and chloramphenicol (Cm), 10 μg/ml when required.

**Table 1:** Bacterial strains and plasmids used in this study

Bacterial strains	Genotype or description	References or sources
*Vibrio parahaemolyticus*		
RIMD2210633	O3:K6 clinical isolate, Sm^r^	[8]
Δ*eda1*	RIMD2210633 Δ*eda1* (VP0065), Sm^r^	This study
UCM-V493	O2:K28 environmental isolate, Sm^r^	[53]
Δ*sglt*	UCM-V493 Δ*sglt* (VPUCM_0844), Sm^r^	This study
RIMDpSGLT	RIMD2210633 harboring pBBR*sglt*, Cm^r^	This study
*Escherichia coli*		
BW25113	Wild-Type *E. coli* K12 strain	[54]
DH5αλpir		Laboratory collection
B2155λpir	*ΔdapA::emr pir*, for bacterial conjugation	Laboratory collection
*Klebsilla pneumoniae* K2		Laboratory collection
*Vibrio cholerae* N16961	O1 El Tor, Sm^r^	[55]
Plasmids		
pJet1.2/blunt	Cloning vector/Amp^r^	Fermentas
Pjet1.2-*edA1*SOEAD	pJet1.2 harboring truncated *eda1*, Amp^r^	This study
pDS132	Suicide plasmid, Cm^r^, SacB	[56]
pDSΔ*eda1*	pDS132 harboring truncated *eda1*, Cm^r^	This study
Pjet1.2-*sglt*SOEAD	pJet1.2 harboring truncated *sglt*, Amp^r^	This study
Pjet1.2-*sglt*	pJet1.2 harboring *sglt*, Amp^r^	This study
pDSΔ*sglt*	pDS132 harboring truncated *sglt*, Cm^r^	This study
pBBR1MCS	Expression vector, lacZ promoter, Cm^r^	[57]
pBBR*sglt*	pBBR1MCS harboring full-length *sglt*, Cm^r^	This study

### Construction of *V. parahaemolyticus* deletion mutants

Splicing by overlapping extension (SOE) PCR and an allelic exchange method [58] was used to construct in-frame, non-polar deletion mutants of VPUCM_0844 (*sglt*) and VP0065 (*eda1*) in *V. parahaemolyticus* strain UCM-V493 and RIMD2210633, respectively. Briefly, primers were designed to the VPUCM_0844 and VP0065 using *V. parahaemolyticus* UCM-V493 and RIMD2210633 genomic DNA as templates. All primers used in this study are listed in Table 2. SOE PCR was conducted to obtain a 117-bp-truncated version of 1632-bp of VPUCM_0844. The *Δsglt* PCR fragment was cloned into the suicide vector pDS132 and named pDS*Δsglt*. pDS*Δsglt* was then transformed into *E. coli* strain β2155 λ*pir*, and was conjugated into *V. parahaemolyticus* UCM-V493. Conjugation was conducted by cross streaking both strains on to LB plate containing 0.3 mM DAP. Bacterial growth lawn from this plate was scrapped, resuspended in LB, serially diluted and plated on LB agar plate containing Sm and Cm. Next day, the colonies were verified for single crossover via PCR. The colonies with single crossover were grown over night in LB supplemented with 3% NaCl with no antibiotic added and were plated onto LB 3% NaCl plate containing 10% sucrose to select for double crossover deletion mutant. The gene deletion was confirmed by PCR. A similar protocol was used to create a deletion of VP0065 (*eda1)*.

**Table 2:** Primers used in this study

Primer name and use	Sequence (5′-3′)^a^	Melting temp (°C)
SOE PCR		
VP0065 SOEA	TCTAGAAACCGTAGCGCTAACCACTA	63
VP0065 SOEB	AATGTCACTTCCGCACATGG	59
VP0065 SOEC	CCATGTGCGGAAGTGACATTTGCAATGATGGATAACGGCG	59
VP0065 SOED	GAGCTCCAGGCCCCAATTTTGAGACC	60
VP0065 SOEFF	GTGTGGTGAGTCATTGGCTC	55
VP0065 SOEFR	GCCAATGCGTACGACAAAGA	50
SGLT SOEA	TCTAGACTTCTACCCTTTGAACTTGCAG	58
SGLT SOEB	GACATAAATGGCGAAGACC	51
SGLT SOEC	GGTCTTCGCCATTTATGTCATCGCTGCTTACGGCATAAT	66
SGLT SOED	GAGCTCGGTTTGTATGCACCACCCAC	63
SGLT SOEFF	GGTGAAAACCACCAAACCTG	55
SGLT SOEFR	TGAGGATGGGCGTTCATAAT	54
Quantitative real-time PCR (qPCR) primers		
VP0065 F	ACCGGGTGTAAACAACCCAA	60
VP0065R	TACCGCCACAAGCTACAACC	60
VP1708F	CACGCCAGTAGAAGGTGACA	60
VP1708R	GTTGCCTAGGAACGCAGAAC	59
VPA0083F	GCAAGGCAGTGAAATTGGCA	60
VPA0083R	ACGTTACGCTGTTGGCAGTA	60
VPA1708F	CAGCCGCTGAAATCACCTTC	55
VPA1708R	TTTCTTGGCACGCTTTGACG	50
16S F	ACGGCCTGGGGAGTACGGTC	60
16S R	TTGCGCTCGTTGCGGGACTT	60
Complementation primers		
SGLTforward	TCTAGAGGATGATCCCCGTAATTTCC	58
SGLTreverse	GAGCTCATTTGGCAGCAAGGTTGAATC	61

^a^Underlined base pairs indicate restriction sites

### Complementation of *V. parahaemolyticus* RIMD2210633 with *sglt* (VPUCM_0844)

RIMD2210633 was complemented with the putative sodium galactose transporter gene *sglt* (VPUCM_0844) from strain UCM-V493 and the resulting strain was designated as RIMDpSGLT. A pair of primers (Table 2) was designed to amplify a copy of VPUCM_0844 (including the endogenous promoter region) from *V. parahaemolyticus* UCM-V493, which was cloned into pJET1.2 and transformed into *E. coli* DH5α λ*pir*. Subsequently, the fragment was subcloned into the vector pBBR1MCS, creating pSGLT, which was transformed into *E. coli* β2155 λ*pir*. pSGLT was transferred into *V. parahaemolyticus* RIMD210633 via conjugation on a LB plate containing 0.3 mM diaminopimelic acid (DAP). The bacterial growth from the conjugation plate was scraped and streaked on LB Sm Cm plates. Colonies were screened to select for *V. parahaemolyticus* RIMD2210633 harboring pSGLT. 1 mM isopropyl β-d-1-thiogalactoside (IPTG) was added to the culture to induce RIMDpSGLT for growth analysis.

### Comparative genomics

Whole genome comparison between the *V. parahaemolyticus* strains was conducted using BLASTn from complete genome sequences downloaded from NCBI bacterial genome database. The genome sequences were visualized by using a Python based application, Easyfig to identify regions homologous to each other and regions unique to each strain [59]. For genes coding hypothetical proteins, putative functions were determined by using HHpred [60]. The Carbohydrate-Active enzymes (CAZymes) database was used to determine the function of many hypothetical proteins (CAZy database: http//www.cazy.org [61].

### CRISPR-Cas analysis

The FASTA files for strains CDC_K5635, 3259, FORC_022 and TUMSAT_H10_H6 were used to identify CRISPR direct repeats and spacers in each sequence using the CRISPRFinder and CRISPRDetect programs [62, 63]. CRISPRtionary program was used to determine how unique each spacer was to each strain and the CRISPRMap program was used to assign type and sub-type to each CRISPR-Cas region [64, 65].

### RNA extractions, cDNA synthesis and qRT-PCR expression analysis

Strains were grown at 37 °C overnight, with aeration, in 5 ml LBS. Cells were then pelleted at 4000 × g for 10 min, washed twice in PBS and resuspended in 5 ml PBS. The resuspended culture was diluted 1:50 into 25 ml of M9 media supplemented with either 10 mM glucose or 10 mM gluconate. Cultures were grown with aeration at 37 °C until late log phase (4 h). RNA was then extracted using TRIzol according to the manufacturer’s instructions (Invitrogen, Carlsbad, CA) according to the manufacturer’s instructions. Total RNA samples were treated with Turbo DNAse (Invitrogen) according to the manufacturer’s instructions. RNA samples were quantified using a Nanodrop spectrophotometer (Thermo-Fisher Scientific, Waltham, MA). cDNA was synthesized with Superscript II reverse transcriptase (Invitrogen) according to the manufacturer’s instructions using 500 ng of RNA as the template and 200 ng of random hexamers in each synthesis reaction. The cDNA samples were diluted 1:50 and used as the template for quantitative real-time PCR (qPCR) using the fast SYBR green master mix (Applied Biosystems) according to the manufacturer’s instructions. Gene primers were designed using Primer 3 and *V. parahaemolyticus* RIMD2210633 genome sequence as the template and are listed in Table 2. Data was analyzed using the ABI 7500 software (Applied Biosciences). The expression levels of each gene, determined by their cycle threshold (CT) values, were normalized using the 16S rRNA gene. Differences in gene expression ratios were determined using the previously described ΔΔCT method [66].

### Phylogenetic analysis

Representative proteins from each metabolism cluster were used as seed sequences in a protein BLAST (pBLAST) to identify putative homologues in *V. parahaemolyticus* as well as in other members of the family *Vibrionaceae*. The phylogenetic trees were constructed from the alignment of the amino acid sequences of the identified proteins of interest. Most of the proteins from each species represent multiple strains with the same protein. The software, Molecular Evolutionary Genetic Analysis version X (MEGA X), was used to infer evolutionary history [29, 40].

### Phenotypic microarray analysis of aerobic growth on 190 carbon sources of RIMD2210633 and UCM-V493

Strains were grown in M9 medium supplemented with 10 mM glucose, overnight, at 37 °C with aeration (250 rpm). Overnight cultures were then diluted 1:50 into 5 ml M9 medium supplemented with 10 mM glucose and grown aerobically for 4 h at 37 °C. Cells were then pelleted at 4000 × g for 10 min and washed twice with PBS. Cells were resuspended in 5 ml PBS and diluted 1:50 into fresh M9 medium with no carbon source and 100 μl was transferred to each well of either a PM1A or PM2A Biolog Phenotypic MicroArray 96 well plate (Biolog, Hayward, CA). Biolog plates were subsequently incubated at 37 °C for 24 h and optical density readings at 595 nm (OD_595_) were taken hourly using a Sunrise Tecan plate reader and Magellan software. Experiments were performed using two biological replicates. To assay the growth patterns of each strain on the various carbon sources, the area under the curve (AUC) over the 24 h of growth was calculated for each carbon source. The first well on each PM plate contained M9 medium plus cells but no carbon source and the area under the curve for this well was used as the blank, which was subtracted from the AUC for each carbon sources to determine which carbon sources *V. parahaemolyticus* exhibited growth. As a cutoff, we considered average AUC below two as no growth. Unpaired student’s t-test was performed using Prism graphpad software to identify significant difference in the AUC (Additional file 1: Table S1).

## Additional files

Additional file 1: **Table S1.** Phenotypic growth analysis of *V. parahaemolyticus* strains RIMD2210633 and UCM-V493. **Table S2.** Pathogenic and non-pathogenic strains of *V. parahaemolyticus* examined in detail in this study. **Table S3.** Completed genomes of *V. parahaemolyticus* strains used for whole genome analysis. **Table S4.** Unique regions (10-kb or >) present in the 16 *V. parahaemolyticus* strains that are absent in RIMD2210633. **Table S5.** *V. parahaemolyticus* strains containing citrate fermentation cluster. **Table S6.** *V. parahaemolyticus* strains containing 26-kb (upper panel) and metabolic island (lower panel) with l-rhamnose cluster. **Table S7.** *V. parahaemolyticus* strains containing d-galactitol, l-arabinose (with MFS transporter), and l-arabinogalactan utilization clusters. (XLSX 49 kb)

Additional file 2: **Figure S1.** Variants of the Metabolic islands containing a citrate fermentation gene cluster in *V. parahaemolyticus*. Gray shade indicates homologous regions between strains. Arrows represent ORFs, ORFs with the same color represent functionally similar proteins. Gray arrows, genes coding hypothetical and other functional proteins. Black arrows represent transposases. **Figure S2.** Variants of the Metabolic island containing l-rhamnose utilization and OGA clusters. A. Comparative analysis of l-rhamnose gene cluster. Gray shade indicates homologous regions between strains. Arrows represent ORFs, identical colored ORFs indicate similar function. Gray arrows, genes coding hypothetical and other functional proteins. Black arrows indicate transposases. B. OGA metabolism pathway with enzymes involved and ORFs identified in the 135-kb metabolic island of FORC_022. OGA catabolism cluster in RIMD2210633 is also shown. uGA_2_, unsaturated galacturonate dimer, GH, glycoside hydrolase. **Figure S3.** Genomic analysis of l-arabinose catabolic gene cluster. A. Comparative analysis region between VPA0441 and VPA0450. Gray shade, region of nucleotide homology B. d-galactitol pathways with proteins and ORFs identified in CFSAN007457. C. l-arabinose gene cluster present in *V. parahaemolyticus* strain RIMD2210633. Arrow indicated ORFs. **Figure S4.** Phylogenetic analysis of AraD among *Vibrionaceae*. AraD from *V. parahaemolyticus* was used as a seed to identify homologues within the *Vibrionaceae*. Most OTUs representing multiple strains. The evolutionary history was inferred using the Neighbor-Joining method [26]. The optimal tree with the sum of branch length = 2.24462315 is shown. The percentage of replicate trees in which the associated taxa clustered together in the bootstrap test (1000 replicates) are shown next to the branches [27]. The evolutionary distances were computed using the Dayhoff matrix based method and are in the units of the number of amino acid substitutions per site [28]. The rate variation among sites was modeled with a gamma distribution (shape parameter = 5). All ambiguous positions were removed for each sequence pair (pairwise deletion option). There were a total of 507 positions in the final dataset. Evolutionary analyses were conducted in MEGA X [29]. **Figure S5.** l-arabinogalactan metabolism cluster within a 62-Kb island. A. A 62-kb island containing l-arabinogalactan catabolic cluster is shown. Arrows indicate ORFs. Gray arrows, genes coding hypothetical and other functional proteins. Black arrow indicates transposase. B. l-arabinogalactan utilization pathway identified in CH25 and ORFs for l-arabinose utilization in RIMD2210633 and FORC_008. **Figure S6.** Predicted model of the 135-kb Metabolic island emergence. The putative progenitor metabolic island was not identified in any strain in the genome databases. Shown is the most evolutionary parsimonious steps required to explain how these variants arose. In all, we identified 10 variants and for the sake of simplicity, we only show five genes clusters from these islands, which also contain restriction modification systems amongst others. (PPTX 7712 kb)

## Abbreviations

HGT: Horizontal gene transfer
MFS: Major facilitator superfamily
SGLT: Sodium galactose transporter
KDG: Keto-deoxy-phosphogluconate
ED: Entner-Doudoroff
PP: Pentose phosphate
OGA: Oligogalacturonide
attL: Attachment site left
attR: Attachment site right
CRISPR: Clustered regularly interspaced short palindromic repeat
Cas: CRISPR associated
R end: Right end
L end: Left end
Tn: Transposon
T3SS: Type three secretion system
PAI: Pathogenicity island
VPaI: *Vibrio parahaemolyticus* island
PTS: Phosphotransferase system
ABC: ATP binding cassette
LB: Lysogeny broth
Sm: Streptomycin
Cm: Chloramphenicol
DAP: Diaminopimelic acid
IPTG: Isopropyl β-d-1-thiogalactoside
SOE: Splicing by overlapping exchange
