# Supplementary figures and images for: Carbohydrate metabolic systems present on genomic islands are lost and gained in Vibrio parahaemolyticus
Source: BMC Microbiol. 2019 May 27;19:112. doi: 10.1186/s12866-019-1487-6 (PMC6537148; doi:10.1186/s12866-019-1487-6)

## Slide 1
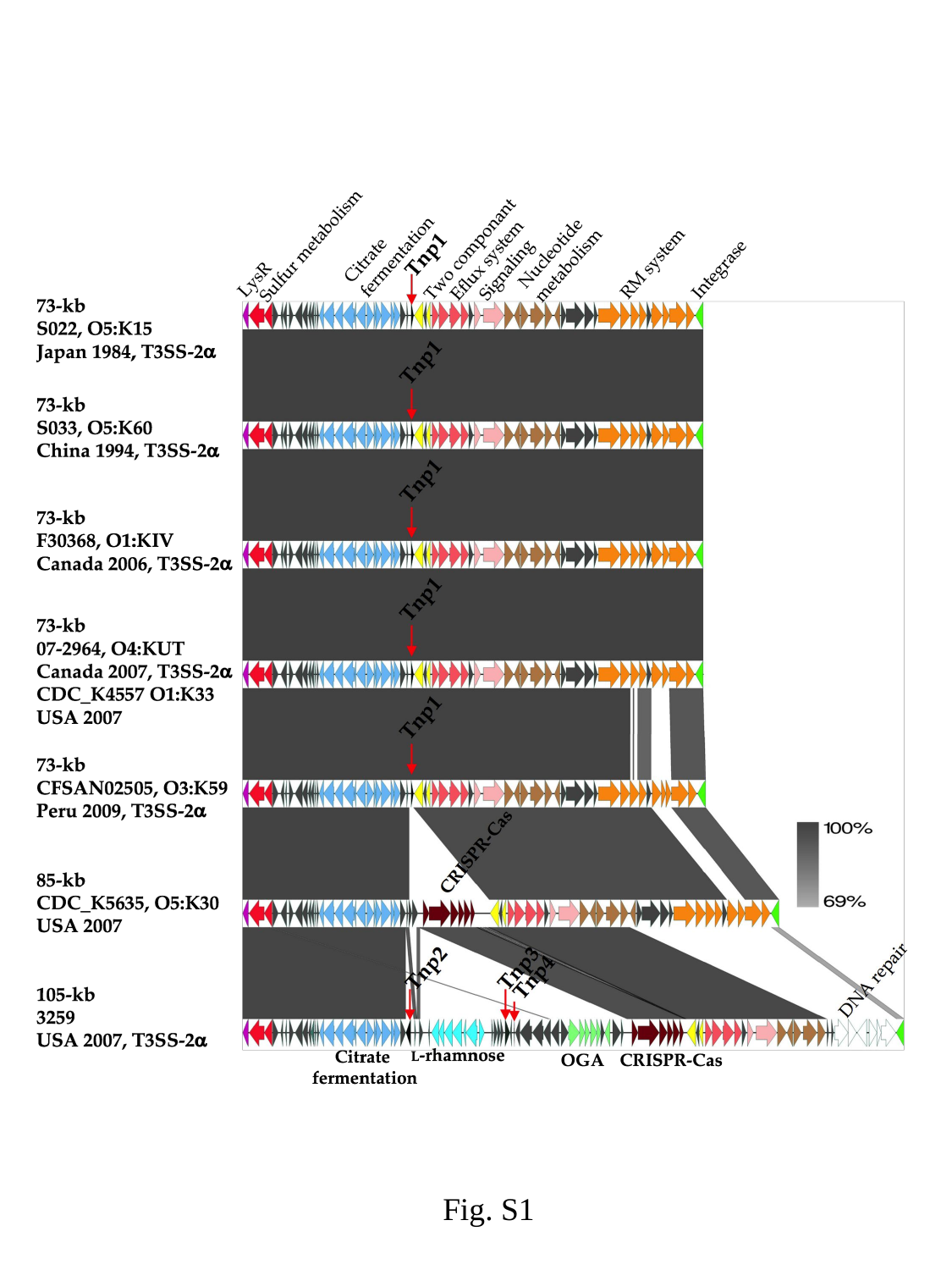

Fig. S1

## Slide 2
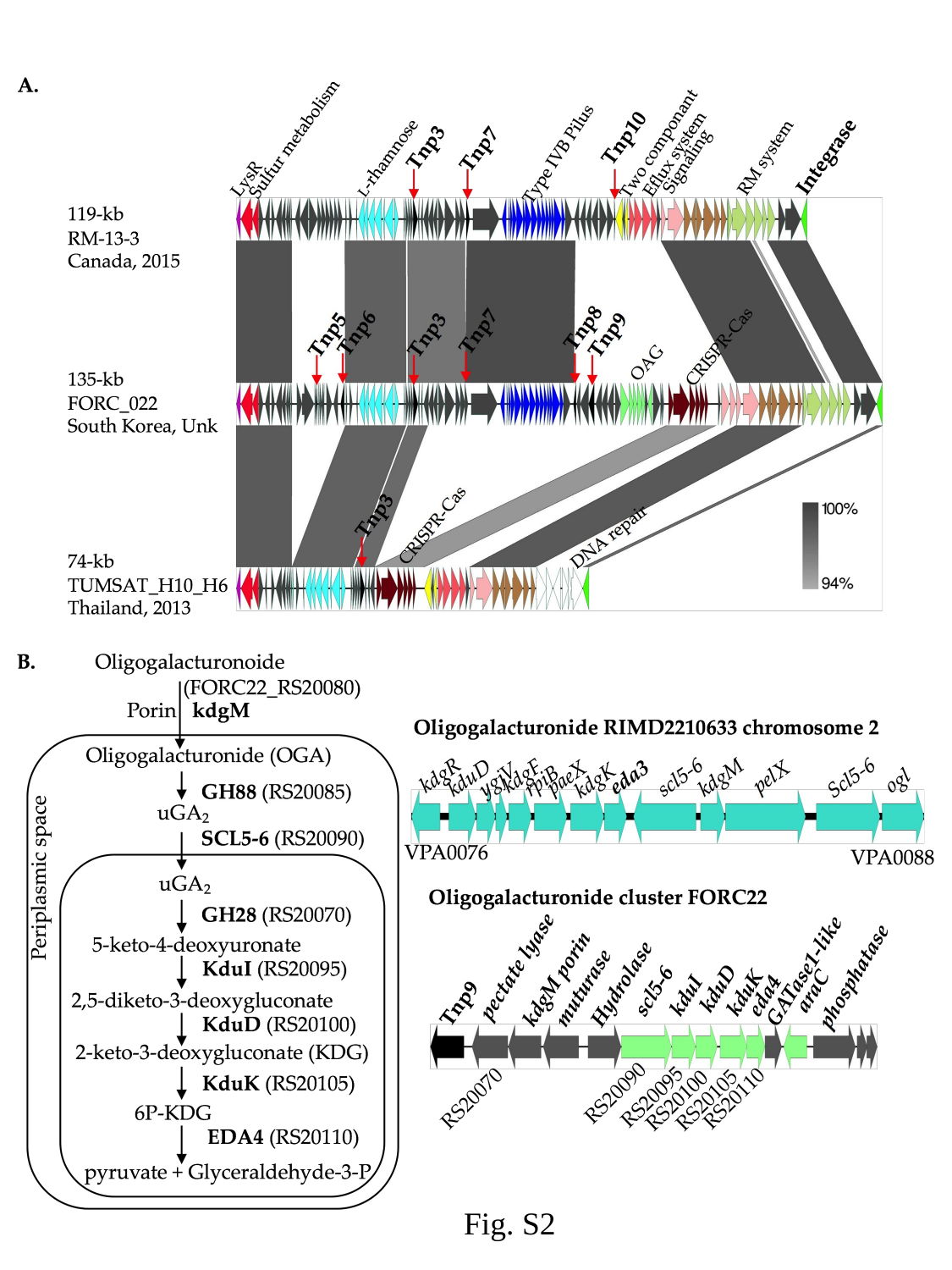

Fig. S2

## Slide 3
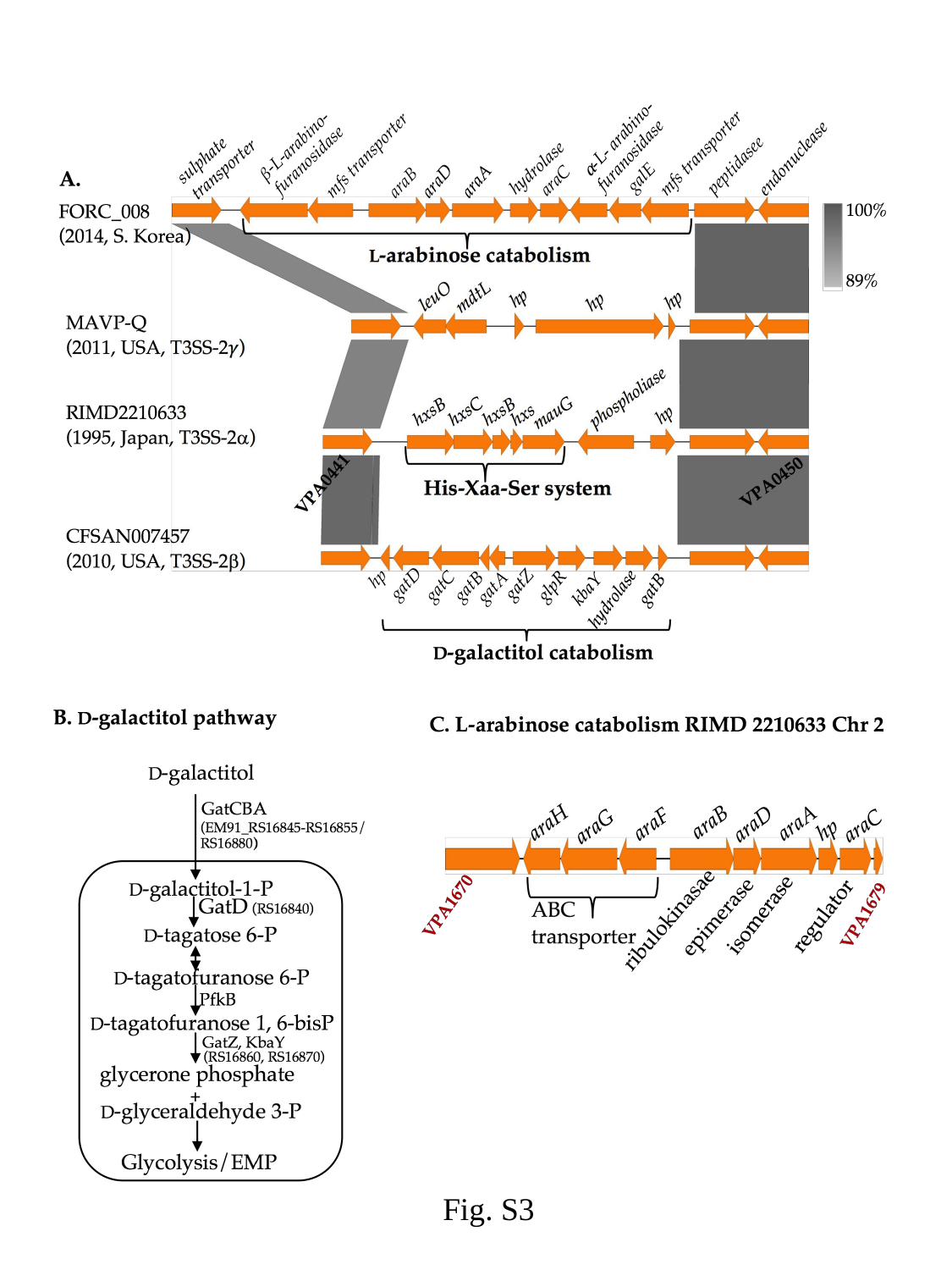

Fig. S3

## Slide 4
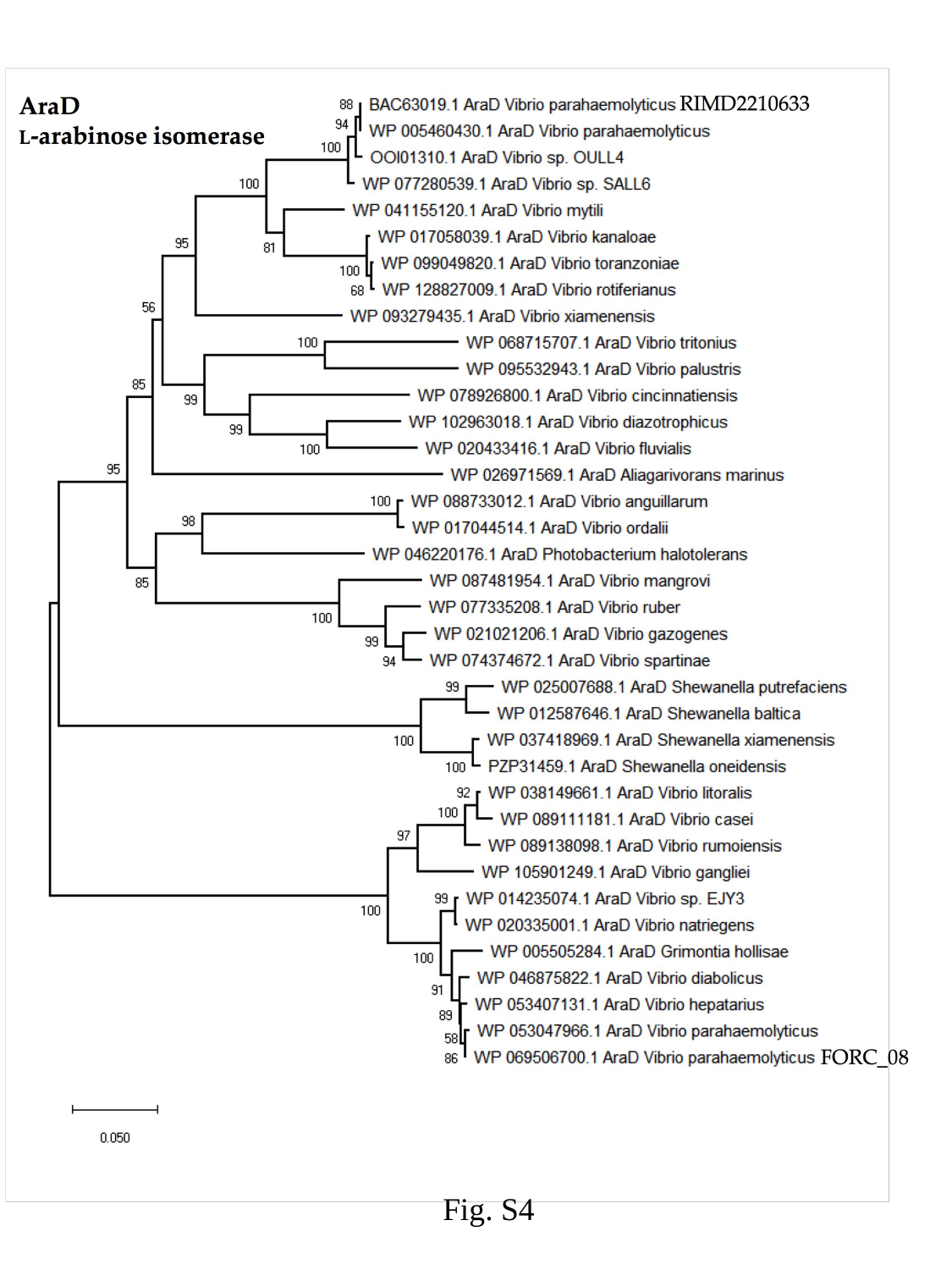

Fig. S4

## Slide 5
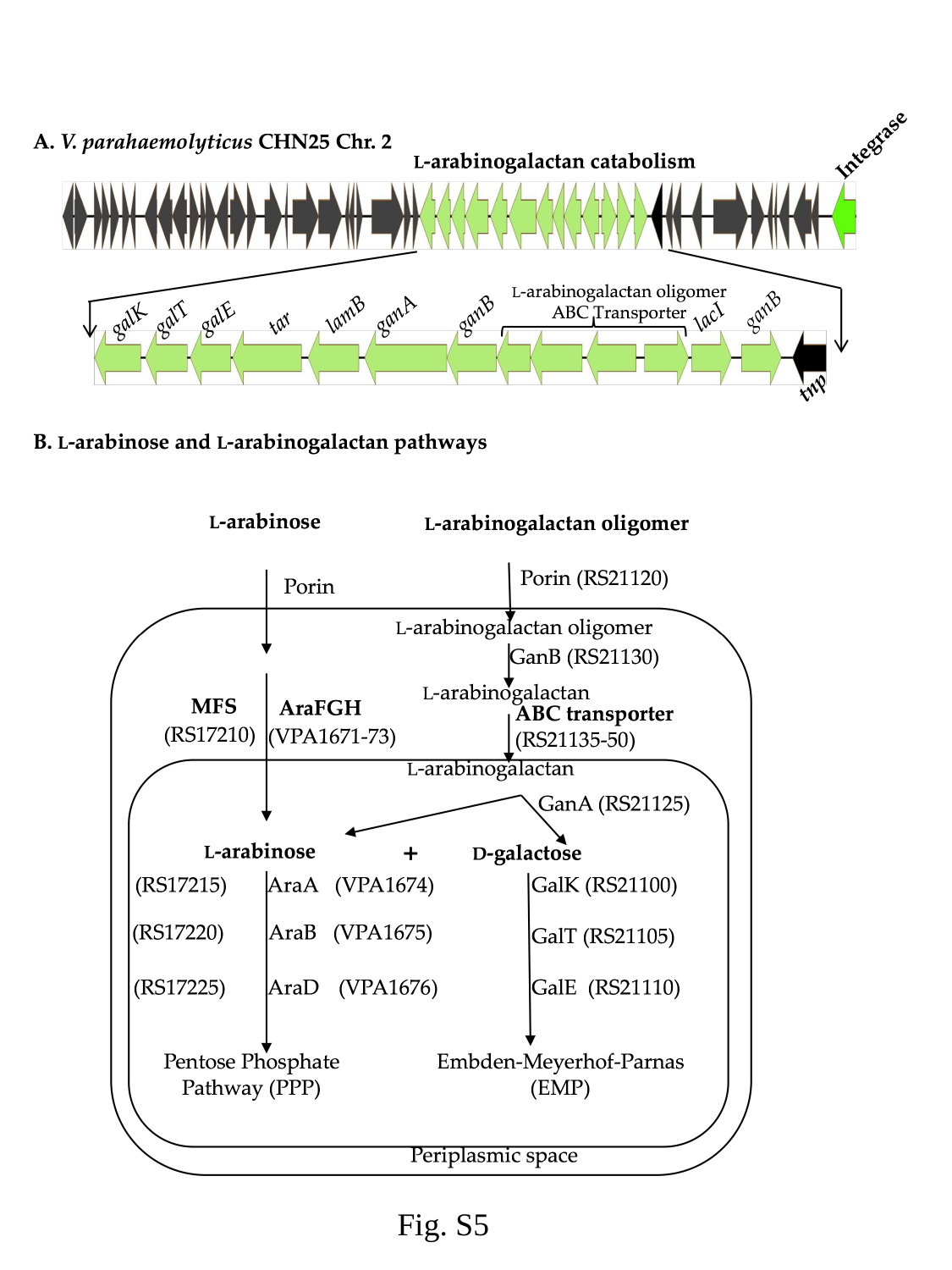

Fig. S5

## Slide 6
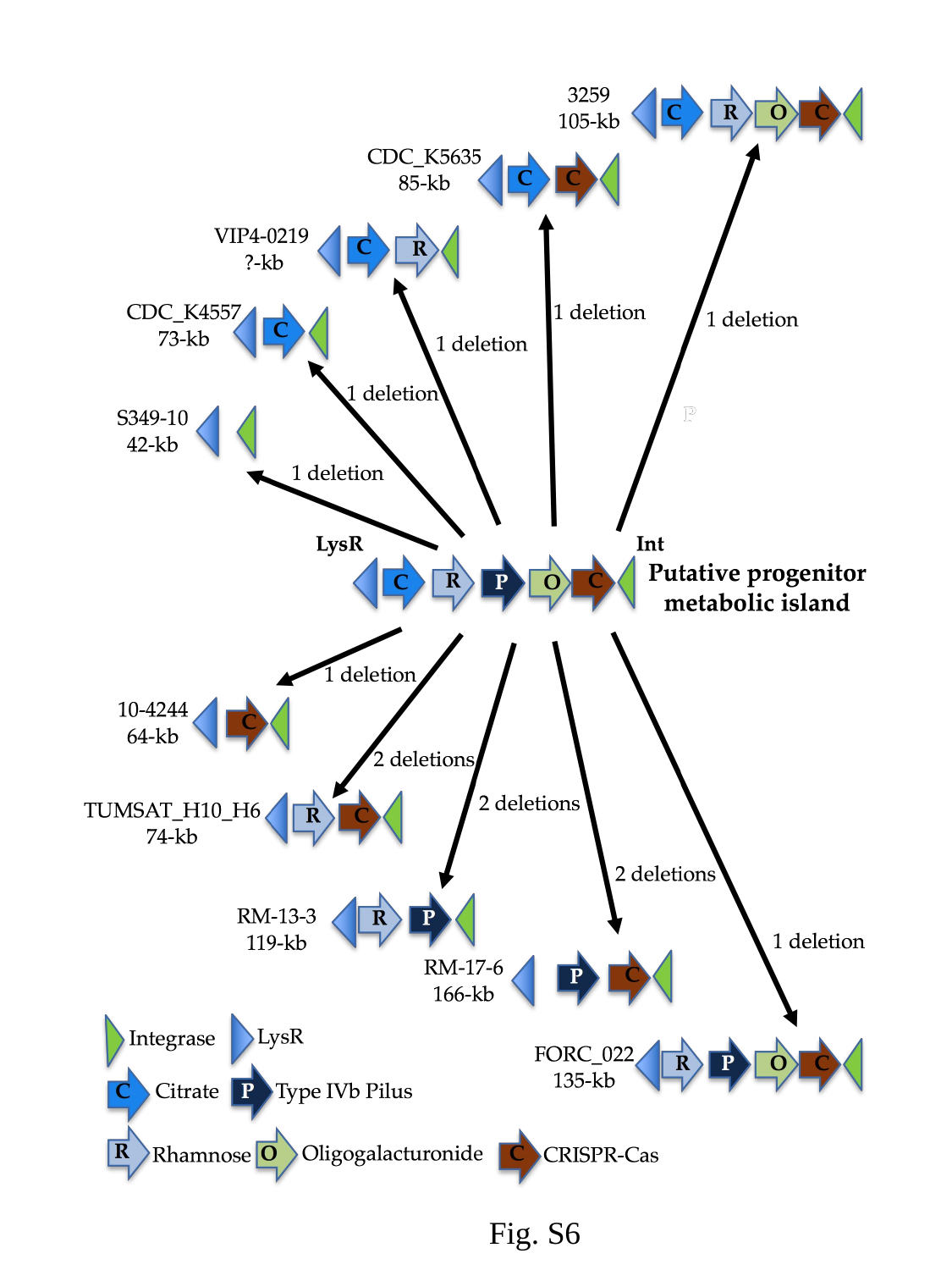

Fig. S6

Supplement: Supplementary file 2 — Figure S1. Variants of the Metabolic islands containing a citrate fermentation gene cluster in V. parahaemolyticus. Gray shade indicates homologous regions between strains. Arrows represent ORFs, ORFs with the same color represent functionally similar proteins. Gray arrows, genes coding hypothetical and other functional proteins. Black arrows represent transposases. Figure S2. Variants of the Metabolic island containing l-rhamnose utilization and OGA clusters. A. Comparative analysis of l-rhamnose gene cluster. Gray shade indicates homologous regions between strains. Arrows represent ORFs, identical colored ORFs indicate similar function. Gray arrows, genes coding hypothetical and other functional proteins. Black arrows indicate transposases. B. OGA metabolism pathway with enzymes involved and ORFs identified in the 135-kb metabolic island of FORC_022. OGA catabolism cluster in RIMD2210633 is also shown. uGA2, unsaturated galacturonate dimer, GH, glycoside hydrolase. Figure S3. Genomic analysis of l-arabinose catabolic gene cluster. A. Comparative analysis region between VPA0441 and VPA0450. Gray shade, region of nucleotide homology B. d-galactitol pathways with proteins and ORFs identified in CFSAN007457. C. l-arabinose gene cluster present in V. parahaemolyticus strain RIMD2210633. Arrow indicated ORFs. Figure S4. Phylogenetic analysis of AraD among Vibrionaceae. AraD from V. parahaemolyticus was used as a seed to identify homologues within the Vibrionaceae. Most OTUs representing multiple strains. The evolutionary history was inferred using the Neighbor-Joining method [26]. The optimal tree with the sum of branch length = 2.24462315 is shown. The percentage of replicate trees in which the associated taxa clustered together in the bootstrap test (1000 replicates) are shown next to the branches [27]. The evolutionary distances were computed using the Dayhoff matrix based method and are in the units of the number of amino acid substitutions per site [28]. The rate v [file 12866_2019_1487_MOESM2_ESM.pptx]
